# Supplementary material for: Sensitive and specific affinity purification-mass spectrometry assisted by PafA-mediated proximity labeling
Source: Cell Rep Methods. 2025 Sep 8;5(9):101166. doi: 10.1016/j.crmeth.2025.101166 (PMC12539243; doi:10.1016/j.crmeth.2025.101166)
Supplement: Document S1. Figures S1–S5 [file mmc1.pdf]

Cell Reports Methods, Volume *5*

Supplemental information

Sensitive and specific affinity purification-mass spectrometry assisted by PafA-mediated proximity labeling

Shihan Luo, Lijuan Xie, Lin Yang, Zheyao Hu, Lei Wang, Yueqin Wang, Qingqing Li, Shujuan Guo, Shengce Tao, and Hewei Jiang

Figure S1

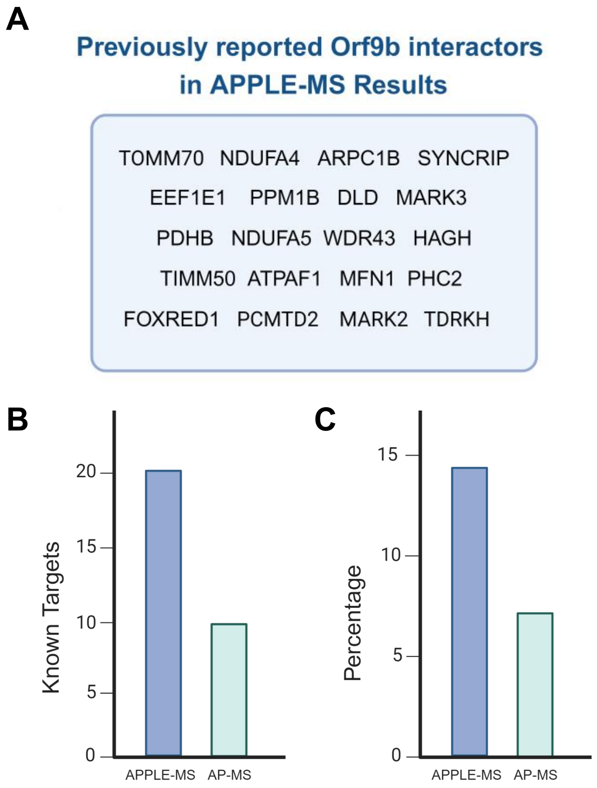

Figure S1 Comparative analysis of ORF9B interactome detection by APPLE-MS versus AP-MS, related to Figure 2.

(A) Reported ORF9B interactors from BioGRID (v4.4.244) detected by APPLE-MS

(B) Quantitative assessment of reported ORF9B interactors. Bar plot illustrates the absolute number of benchmark ORF9B interactors identified by APPLE-MS (blue) versus conventional AP-MS (green).

(C) Detection efficiency for reported ORF9B interactors. Bar plot shows the percentage of known interactors among total identified putative binding partners for each method.

Figure S2

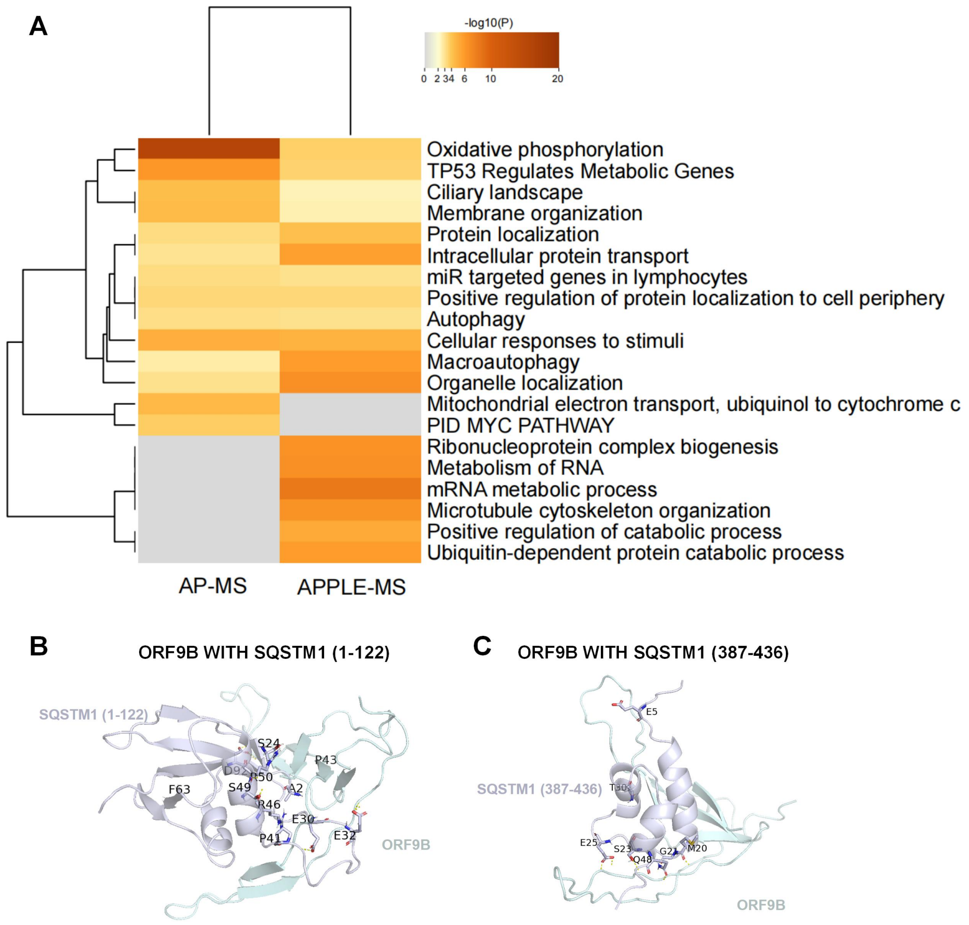

Figure S2 Comparative interactome analysis and structural modeling of ORF9B~ SQSTM1 complexes, related to Figure 3.

(A) GO enrichment comparison of ORF9B interactors between AP-MS (left) and APPLE-MS (right). Color intensity indicates -log10(p value).

(B) and (C) Predicted interaction interfaces between ORF9B and SQSTM1. Molecular dynamics simulations of ORF9B (cyan) binding to distinct SQSTM1 domains (purple).

Figure S3

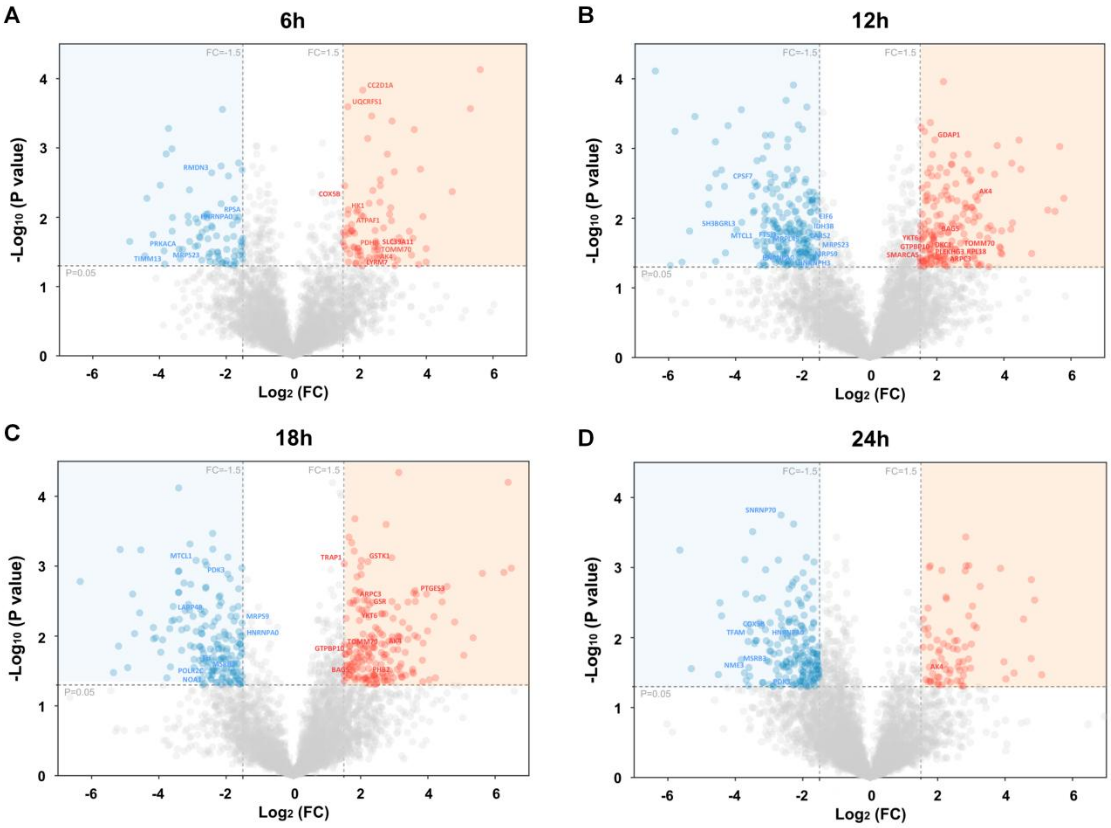

Figure S3 Temporal Profiling of the ORF9B Interactome Following Poly(I:C) Transfection, related to Figure 4.

(A-D) Time-resolved analysis of ORF9B-protein interactions after poly(I:C) transfection. Volcano plots display significantly enriched interactors ($p < 0.05$, fold-change > 2) at each time point (6, 12, 24, and 48 h; n = 3 biological replicates). Literature-curated ORF9B interactors from BioGRID (v4.4.244) are highlighted in red. Dashed lines indicate statistical thresholds (horizontal: p = 0.05; vertical: 1.5-fold change).

Figure S4

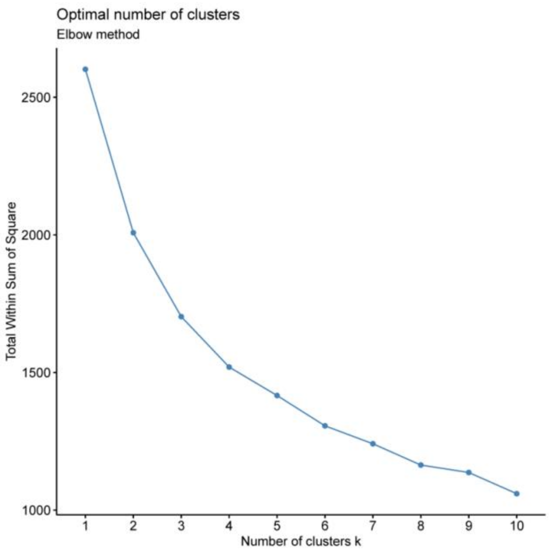

Figure S4 Determination of Optimal Cluster Number in Mfuzz Analysis, related to Figure 4.

Line plot of SSR values versus cluster number (k = 1–10) computed from Mfuzz soft clustering of ORF9B interactome dynamics (0–24 h poly(I:C)). The elbow point at k = 4 was selected as the optimal cluster number, balancing model fit and biological interpretability.

Figure S5

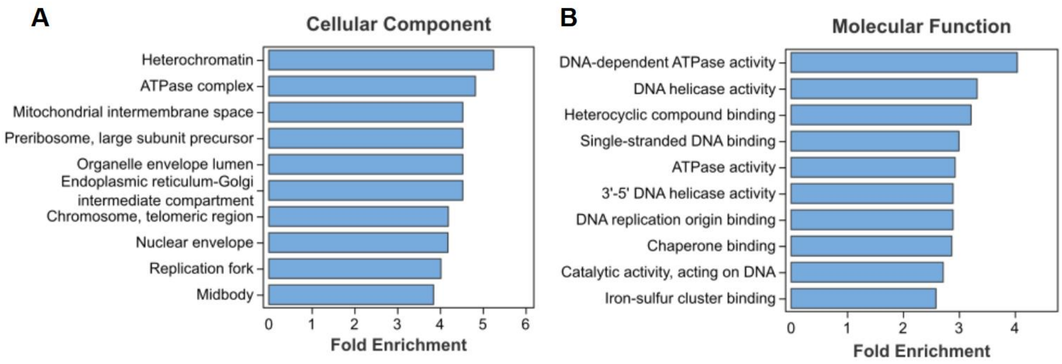

Figure S5 Cellular component and molecular function of PIN1 putative interactors, related to Figure 5.

(A) Subcellular localization of ORF9B interactome. Bar plot shows significantly enriched cellular components (FDR<0.05, hypergeometric test) for ORF9B-binding partners.

(B) Molecular functions enriched in ORF9B interactors. Bar plot illustrates significantly enriched molecular functions (FDR<0.05, hypergeometric test) among ORF9B-associated proteins identified by APPLE-MS.

In (A) and (B), the length of each bar corresponds to the $-\log_{10}$(FDR) value, representing the statistical significance of enrichment.
