# Supplementary material for: Sensitive and specific affinity purification-mass spectrometry assisted by PafA-mediated proximity labeling
Source: Cell Rep Methods. 2025 Sep 8;5(9):101166. doi: 10.1016/j.crmeth.2025.101166 (PMC12539243; doi:10.1016/j.crmeth.2025.101166)
Supplement: Document S2. Article plus supplemental information [file mmc8.pdf]

Cell Reports Methods

Article

Sensitive and specific affinity purification-mass spectrometry assisted by PafA-mediated proximity labeling

Graphical abstract

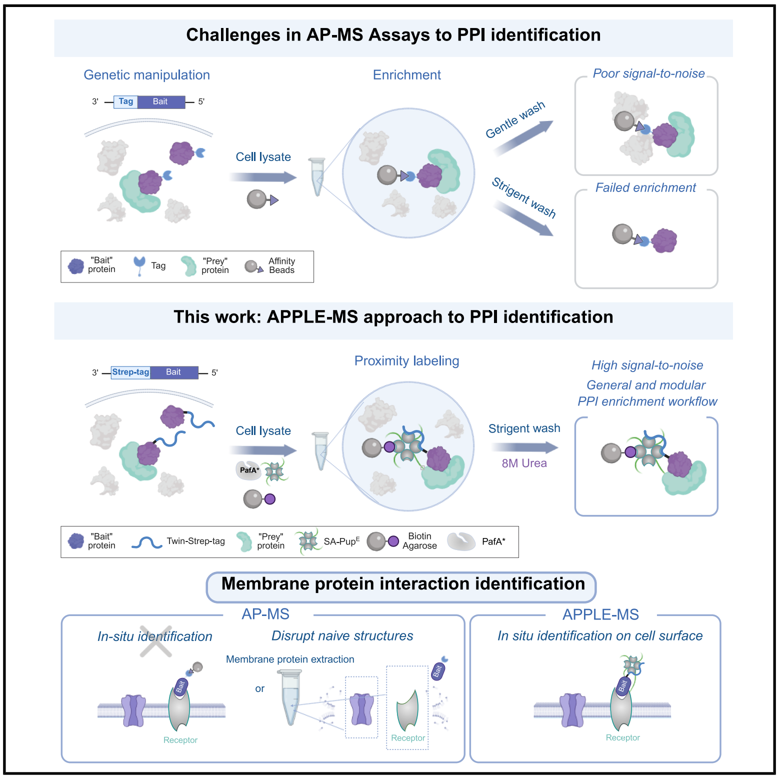

Highlights

- Combined Twin-Strep tag enrichment and PafA-mediated labeling enhances PPI detection
- APPLE-MS captures weak/transient interactions and membrane PPIs in native contexts
- APPLE-MS reveals dynamic SARS-CoV-2 ORF9B interactome during antiviral responses
- APPLE-MS enables endogenous PIN1 interactome mapping with minimal tag interference

Authors

Shihan Luo, Lijuan Xie, Lin Yang, ..., Shujuan Guo, Shengce Tao, Hewei Jiang

Correspondence

taosc@sjtu.edu.cn (S.T.),
jianghewei@lglab.ac.cn (H.J.)

In brief

Luo et al. introduce APPLE-MS, a method integrating affinity purification and PafA-mediated proximity labeling to overcome limitations in detecting weak, transient, and membrane-associated protein interactions. The approach reveals dynamic viral-host interactomes and endogenous protein networks, offering broad applications in interactome research and therapeutic target discovery.

Luo et al., 2025, Cell Reports Methods 5, 101166
September 15, 2025 © 2025 The Author(s). Published by Elsevier Inc.
https://doi.org/10.1016/j.crmeth.2025.101166

CellPress

Cell Reports Methods

CellPress
OPEN ACCESS

Article

Sensitive and specific affinity purification-mass spectrometry assisted by PafA-mediated proximity labeling

Shihan Luo,[1,2,5] Lijuan Xie,[2,5] Lin Yang,[3,5] Zheyao Hu,[1,5] Lei Wang,[1,2] Yueqin Wang,[4] Qingqing Li,[2] Shujuan Guo,[1] Shengce Tao,[1,*] and Hewei Jiang[2,6,*]

[1]Shanghai Center for Systems Biomedicine, Key Laboratory of Systems Biomedicine (Ministry of Education), Shanghai Jiao Tong University, Shanghai 200240, China

[2]Lingang Laboratory, Shanghai 201306, China

[3]Institute of Neuroscience, Chinese Academy of Sciences, Shanghai 200031, China

[4]Shanghai Key Laboratory of New Drug Design, School of Pharmacy, East China University of Science and Technology, Shanghai 200237, China

[5]These authors contributed equally

[6]Lead contact

*Correspondence: taosc@sjtu.edu.cn (S.T.), jianghewei@lglab.ac.cn (H.J.)

https://doi.org/10.1016/j.crmeth.2025.101166

MOTIVATION Affinity purification-mass spectrometry (AP-MS) has become a widely used method for capturing high-affinity protein-protein interactions (PPIs). However, it still faces several limitations, including high levels of non-specific binding, challenges in detecting weak interactions, and limited ability to identify PPIs at the cell surface *in situ*. To overcome these challenges, we introduce a modular AP-MS method enhanced by PafA-mediated proximity labeling, which improves both the specificity and sensitivity of PPI detection in a single, streamlined workflow.

SUMMARY

While affinity purification-mass spectrometry (AP-MS) has significantly advanced protein-protein interaction (PPI) studies, its limitations in detecting weak, transient, and membrane-associated interactions remain. To address these challenges, we introduced a proteomic method termed affinity purification coupled proximity labeling-mass spectrometry (APPLE-MS), which combines the high specificity of Twin-Strep tag enrichment with PafA-mediated proximity labeling. This method achieves improved sensitivity while maintaining high specificity (4.07-fold over AP-MS). APPLE-MS also revealed the dynamic mitochondrial interactome of severe acute respiratory syndrome coronavirus 2 (SARS-CoV-2) ORF9B during antiviral responses, while endogenous PIN1 profiling uncovered novel roles in DNA replication. Notably, APPLE-MS enabled *in situ* mapping of GLP-1 receptor complexes, demonstrating its unique capabilities for membrane PPI studies. This versatile method advances interactome research by providing comprehensive, physiologically relevant PPI networks, opening new opportunities for mechanistic discovery and therapeutic targeting.

INTRODUCTION

Protein-protein interactions (PPIs) underpin almost every biological pathway, with more than 80% of proteins functioning within complexes to execute their roles in cells.[1] Dissecting PPIs is therefore essential not only for deciphering molecular mechanisms but also for identifying potential therapeutic targets. However, PPIs are inherently complex and dynamic, influenced by post-translational modifications (PTMs), cellular context, and transient binding events.[2] Over the years, numerous methods have been developed to tackle these challenges, ranging from yeast two-hybrid (Y2H) screens to advanced computational predictions.[3–5] Despite this diversity, each approach faces technical constraints, such as high false-positive rates, suboptimal detection sensitivity, or limited utility in detecting weak or transient interactions.

Affinity purification-mass spectrometry (AP-MS) is one of the most widely adopted methods for defining PPIs in biology laboratories owing to its established protocols and broad applicability, and its integration with quantitative proteomics has facilitated large-scale network studies.[6] However, a persistent challenge lies in the high sensitivity to operational details; small

 © 2025 The Author(s). Published by Elsevier Inc.
This is an open access article under the CC BY license (http://creativecommons.org/licenses/by/4.0/).

variations in execution often lead to inconsistent results among different operators. Additionally, the risk of non-specific binding remains a significant drawback. Tandem AP partially addresses this issue by introducing sequential purification steps; however, the stringent washes involved can inadvertently remove weak interactions, creating a trade-off between sensitivity and specificity.[7] AP-MS has also been used to analyze cell-surface PPIs by introducing affinity tags prior to cell lysis or by detergent-based membrane protein extraction. However, pre-lysis labeling methods frequently induce artificial receptor clustering through harsh washing conditions(e.g., low-pH elution),[8] while detergent-based extraction protocols disrupt critical membrane microdomains and lead to significant loss of peripheral interactors. For example, LPG14, a commonly used anionic detergent, has been shown to dissociate up to 75% of interacting partners from NBD1, underscoring the inherent compromise between effective solubilization and maintaining native interactions.[9] These technical constraints lead to significant underrepresentation of biologically relevant but labile interactions, particularly transient complexes ($t_{1/2}$ < 2 min) and low-affinity assemblies (K_D >100 μM) that dominate cell signaling processes. Proximity labeling (PL)-MS offers a complementary route to map protein networks.[10] By harnessing engineered enzymes or chemical probes that label proximal proteins, PL-MS excels at capturing weak or transient associations, facilitating the discovery of novel PPIs. In workflows such as BioID or APEX, AP with streptavidin beads is integral for enriching the labeled proteins, enabling high-confidence identification via subsequent MS. Although sophisticated strategies combining BioID and AP-MS into a single workflow have been reported,[11] a major caveat is the need to fuse the labeling enzyme (typically 20–50 kDa) to the protein of interest. The introduction of additional mass may disrupt proper protein folding or molecular interactions, and the insertion of such an enzyme at the endogenous locus poses significant technical challenges compared to endogenous AP-MS, which requires only a small epitope tag.

Here, we introduce AP-coupled PL-MS (APPLE-MS), a proteomic interactome profiling method that combines the convenience of AP-MS with the covalent capture of transient interactions afforded by PL. By exploiting the proximity-dependent enzymatic activity of PafA to label neighboring proteins covalently with Pup^E, our approach captures PPIs in their native cellular context. The strong binding affinity between the Twin-Strep tag and streptavidin facilitates efficient enrichment and subsequent MS analysis. This design yields high sensitivity for detecting low-abundance or transient interactions and, crucially, permits *in situ* identification of cell-surface PPIs. We validated APPLE-MS by systematically interrogating intracellular and extracellular PPIs, highlighting its ease of operation, adaptability, and reliability in uncovering interaction networks across distinct cellular compartments.

RESULTS

APPLE enables the detection of PPIs

PafA, a central enzyme in the prokaryotic ubiquitin-like protein (Pup) tagging system, has been demonstrated in multiple studies to exhibit PL activity by catalyzing the ATP-dependent covalent attachment of the C-terminal glutamate residue of Pup^E to lysine side chains on target proteins.[12] Multiple studies have demonstrated that PafA exhibits robust PL activity, enabling the covalent ligation of Pup^E to lysine residues on proximal interactors. Leveraging this proven PL capability and the high affinity between the Twin-Strep tag and streptavidin,[13] we designed APPLE-MS to identify PPIs (Figure 1A). In this approach, a Twin-Strep tag is fused to the bait protein, enabling its efficient capture by streptavidin. Upon addition of PafA and streptavidin-Pup^E (SA-Pup^E), Pup^E is covalently attached to neighboring proteins, facilitating subsequent MS-based identification of interactors. This method isolates PPIs under stringent washing conditions through the high-affinity biotin-streptavidin interaction, allowing for efficient purification of prey proteins and the capture of PPIs in a single integrated workflow.

To benchmark APPLE-MS and empirically determine its detectable affinity range, we tested it against a well-characterized model system (Figure 1B). The dimeric meprin and TRAF homology (MATH) domain of speckle-type POZ protein (SPOP) recognizes small peptides containing distinct motifs via conserved hydrophobic and polar interactions, with sequence variations among substrates leading to differences in binding affinity.[14] Peptide 1 demonstrates the highest binding affinity for the MATH domain (3.7 μM), followed by peptide 2 (76 μM) and peptide 3 (>1,000 μM), reflecting diminishing affinities due to alterations in their SBC (SPOP-binding consensus) motif sequences. We genetically fused the Twin-Strep tag to each peptide and performed an APPLE-MS assay using the GFP-tagged MATH domain as the binding substrate. Our method successfully detected the SA-Pup^E–MATH complex for peptide 1 and peptide 2 (Figure 1C), demonstrating the ability of APPLE-MS to capture weak interactions with affinities of at least 76 μM. Furthermore, increasing concentrations of free peptide 1 progressively inhibited MATH modification by outcompeting the Twin-Strep-tagged version in the APPLE-MS assay (Figure 1D). Altogether, these features enable APPLE-MS to robustly capture both stable and weak interactions.

APPLE-MS to identify the ORF9b interactome

To further validate the applicability of APPLE-MS for systematic PPI screening, we employed this approach to comprehensively characterize the interactome of severe acute respiratory syndrome coronavirus 2 (SARS-CoV-2) ORF9B. As a principal immune evasion factor, ORF9B suppresses host innate immunity by interacting with the mitochondrial translocase receptor TOM70, thereby inhibiting the recruitment of Hsp90 and associated signaling proteins, which impairs the interferon response.[15,16] However, its complete interactome remains poorly characterized, primarily due to limited sensitivity of existing methods (e.g., AP-MS) in detecting weak or transient interactions. These limitations prompted us to select ORF9B as an ideal model for mapping the APPLE-MS-based interactome, leveraging the method's sensitivity to capture both stable immune regulators and weak viral-host interactions (Figure 2A).

To specifically target interactors, we engineered a C-terminally Twin-Strep-tagged ORF9B construct (ORF9B-Twin-Strep tag) for comprehensive interaction profiling. Immunofluorescence microscopy showed strong mitochondrial localization of

CellPress
OPEN ACCESS

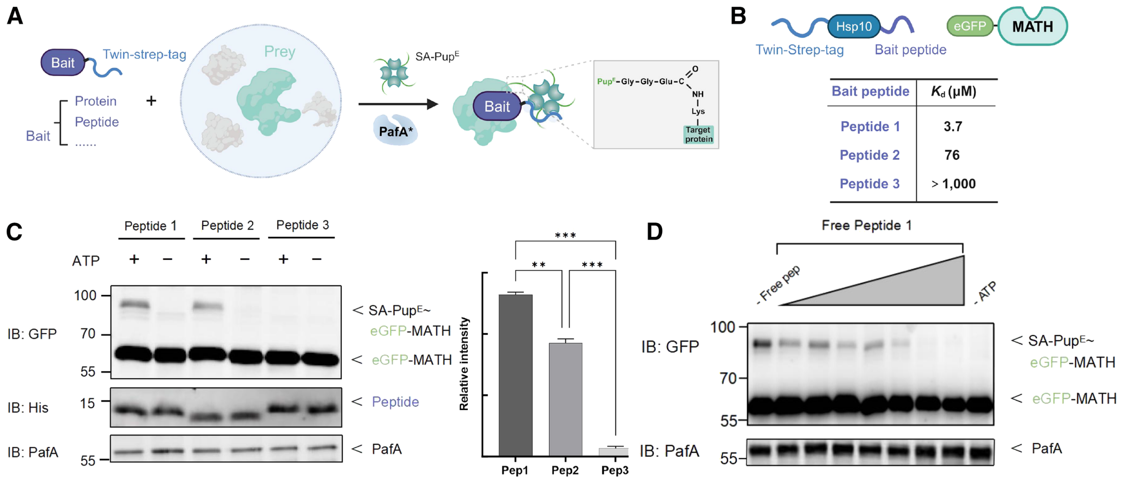

Figure 1. Design and validation of the APPLE PL system

(A) Schematic of the APPLE PL system. The bait protein (purple) is fused to a Twin-Strep tag (blue) for streptavidin-Pup^E (SA-Pup^E) recruitment. PafA mediates covalent Pupylation of proximal lysine residue(s) on the prey protein (green), converting the transient interaction between the bait and prey into stable SA-Pup^E-prey conjugates.

(B) Model system for evaluating the affinity detection limit in the APPLE assay. Three Twin-Strep-tagged peptides (1–3) with MATH domain binding affinities ranging from 10^3 to 10^6 M^{-1} were designed. Binding affinity to the MATH domain decreases sequentially from peptide 1 to peptide 3.

(C) Quantitative validation of the affinity detection limit. Shown is the APPLE assay with purified GFP-tagged MATH and affinity-varied peptides. Data represent mean ± SEM from three independent experiments. $^{**}p < 0.01$ and $^{***}p < 0.001$ (two-tailed unpaired t test).

(D) Competition binding assay. Shown is dose-dependent inhibition of Twin-Strep-tagged peptide 1 binding by untagged peptide 1 in the APPLE system.

ORF9B-Twin-Strep tag, with significant colocalization observed for the mitochondrial import receptor TOMM70 (also known as TOM70) (Figure 2B). This observation aligns with previous studies identifying TOMM70 as a canonical binding partner of ORF9B.[16] Next, we co-expressed ORF9B-Twin-Strep tag with Myc-tagged TOMM70 in HEK293T cells and performed an APPLE-MS assay. As expected, the ORF9B-TOMM70 interaction mediated efficient SA-Pup^E binding to Myc-TOMM70, confirming the proximity-dependent labeling capability of our APPLE-MS platform (Figure 2C).

We then applied APPLE-MS to systematically characterize the ORF9B interactome, identifying 138 high-confidence interactors (Figure 2D). To benchmark performance, we conducted parallel analyses using conventional AP-MS with Twin-Strep tag enrichment via Streptactin beads (Figure 2E). Comparative analysis (Table S1) revealed three key advantages of APPLE-MS. First, it achieved a 4.07-fold improvement in the number of identified interactors relative to the total number of detected proteins, and it identified twice as many literature-curated interactors from the BioGRID database compared to conventional AP-MS (Figure S1). Second, whereas conventional AP-MS detected an average of 7,353 proteins per group, our approach confidently identified 4,196 unique proteins per group, with an increased proportion of high-confidence interactors, demonstrating more effective enrichment. Third, APPLE-MS significantly enhanced the signal-to-noise ratio in interaction detection (Table S2). Here, "noise" was defined as data points meeting all of the following criteria: (1) $p > 0.05$, (2) detection in $\geq$2 blank controls (untransfected lysates), (3) high inter-replicate variability in both experimental and control groups (Coefficient of variation > 30%), and (4) absence from the traditional MS contamination protein list (CRAPome[17]). This side-by-side comparison enabled a direct assessment of APPLE-MS's distinct advantages in capturing weak interactions, underscoring its capacity to detect both canonical and novel interactions with high specificity.

We further evaluated each method's ability to characterize protein complexes associated with ORF9B. Both AP-MS and APPLE-MS identified not only ORF9B's direct interactors but also secondary complex components, with APPLE-MS providing superior coverage of indirect associations reported in BioGRID (Figure 2G). This improved coverage highlights APPLE-MS's enhanced ability to map extended protein interaction networks beyond primary interactors.

To gain mechanistic insight into the biological roles of these identified interactors, we performed Gene Ontology (GO) and Kyoto Encyclopedia of Genes and Genomes (KEGG) analyses (Figures 3A–3D). The results support ORF9B's roles in viral pathogenesis by revealing its involvement in mitochondrial processes (e.g., oxidoreductase activity), immune regulation, and host mRNA processing, consistent with published evidence that ORF9B localizes to mitochondria and antagonizes interferon signaling.[16] Notably, the enrichment of P bodies and spliceosomal complexes corroborates ORF9B's documented capacity to hijack host mRNA processing machinery to suppress translation.[18] These RNA-related pathways further suggest that ORF9B manipulates nucleic acid metabolism to promote viral replication. Moreover, the biological processes and molecular functions identified by APPLE-MS complement those revealed by AP-MS, together offering a more comprehensive interaction profile (Figure S2). While AP-MS effectively pinpoints stable protein complexes involved in oxidative phosphorylation, APPLE-

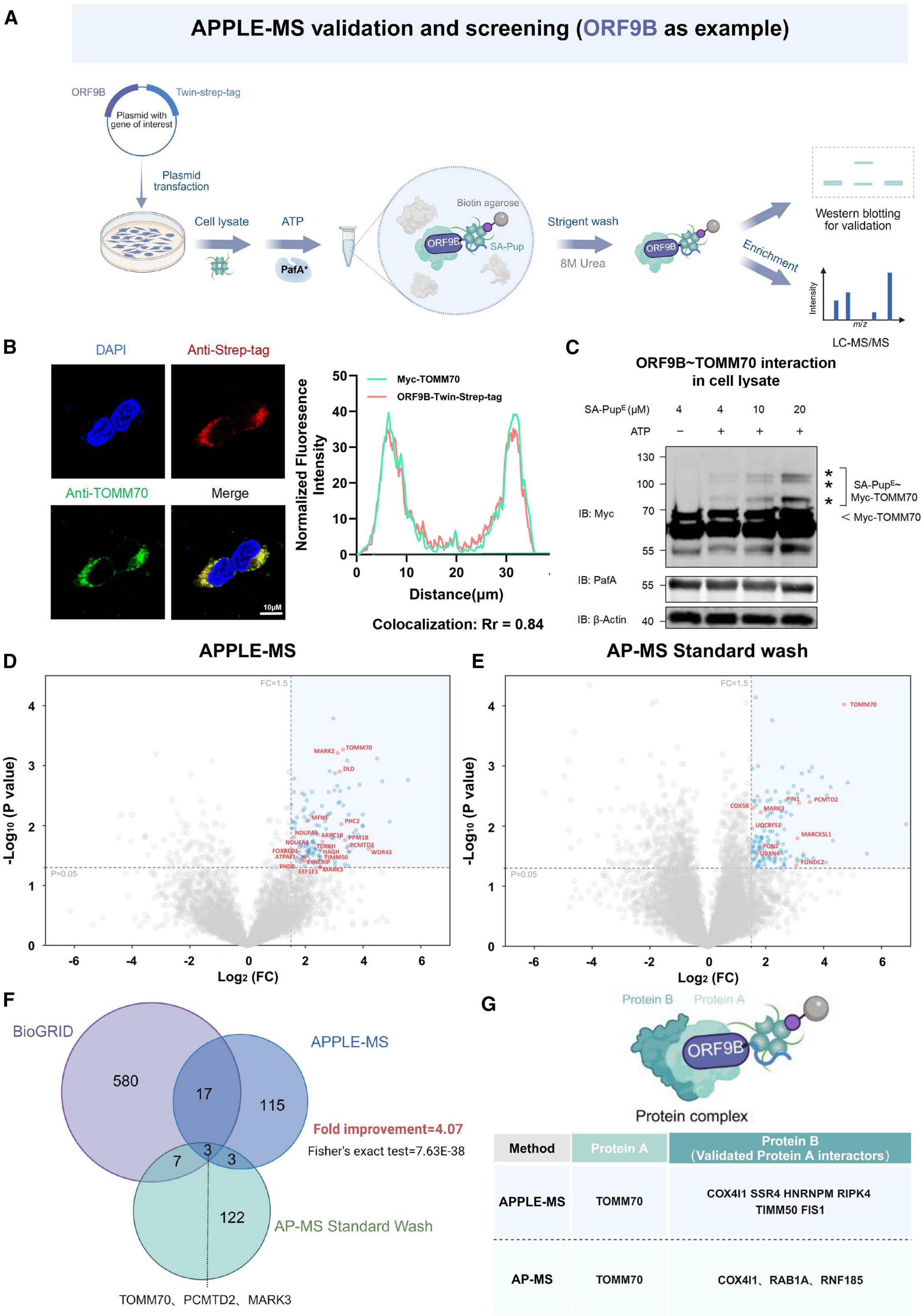

(legend on next page)

MS demonstrates superior performance in capturing metabolic and transcriptional pathways.

Finally, we constructed a PPI network of ORF9B from APPLE-MS data (Figure 3E). MCODE analysis identified three high-confidence modules (scores≥4.0), implicating ORF9B in modulating host translation machinery, peroxisomal protein import, and transport of mature transcripts to the cytoplasm. Notably, 11 of the 17 module members are known regulators of RNA processing and ribosome biogenesis, including RBM19 and RAVER1, which we prioritized for experimental validation. Co-immunoprecipitation (coIP) assays confirmed five ORF9B interactors—KNSTRN, RBM42, SQSTM1, RAVER1, and RBM19 (Figure 3F)—whose functional roles align with the predicted modules. Specifically, RBM42 regulates translation and splicing during DNA damage,[19] SQSTM1 mediates peroxisome autophagy,[20] and RAVER1 contributes to transcript processing.[21] In addition, molecular docking generated high-confidence structural models of these interactions (Figure 3G), highlighting conserved interfacial residues that may stabilize the complexes. Together, these findings demonstrate the reliability of APPLE-MS-identified protein targets and validate the robustness of this method in PPI detection.

APPLE-MS to identify the dynamic interactome of ORF9B

To demonstrate the applicability of APPLE-MS for capturing dynamic PPIs, we employed this approach to systematically characterize the interactome of SARS-CoV-2 ORF9B. By leveraging the superior sensitivity of APPLE-MS, we aimed to explore how ORF9B interacts with host proteins over time and in response to viral immune activation. To achieve this, we used poly(I:C), a synthetic analog of double-stranded RNA, which activates pattern recognition receptors, notably RIG-I and MDA5, to trigger potent intracellular antiviral immune responses.[22] As a well-established mimic of viral RNA, poly(I:C) effectively recapitulates key antiviral defense mechanisms, making it a crucial tool for studying host responses to RNA viruses. Leveraging the superior sensitivity of APPLE-MS, we systematically characterized the dynamic interactome of SARS-CoV-2 ORF9B during a 24-h poly(I:C) stimulation time course in HEK293T cells (Figure 4A). This experimental design was guided by the observed temporal upregulation of RIG-I expression (Figure 4B), which served as a marker of successful immune activation.

We identified 104 interactors of ORF9B that were consistently detected across both initial screening and time-course experiments. Analysis of the MS data (Figure S3; Table S3) revealed significant temporal shifts in the ORF9B interactome under antiviral conditions. Notably, we observed enhanced interactions between ORF9B and critical components of the RIG-I/DDX58-MAVS signaling cascade, such as the E3 ubiquitin ligase RNF123 and mitochondrial translation elongation factor TUFM. We also identified novel associations with key regulators of apoptosis and autophagy pathways, including casein kinase CSNK2A1 and DNA damage checkpoint kinase CHEK2. Additionally, ORF9B interacted with metabolic regulators, such as the lipid droplet protein PLIN2 and glycosyltransferase POMGNT1 (Figures 4C and 4D). In contrast, ORF9B showed reduced binding affinity for the core proteasomal subunits PSMD12/13 and the ubiquitin-like modifier PLAA, suggesting an active evasion of host protein quality control systems. These findings highlight ORF9B as a multifunctional virulence hub, capable of modulating host immunity through ubiquitination and phosphorylation networks, reprogramming cellular metabolism, evading proteasomal degradation, and promoting viral replication.

To comprehensively characterize the temporal dynamics of ORF9B interactions, we employed Mfuzz,[23] a soft clustering algorithm for time-series data, to categorize ORF9B interactors into four distinct clusters based on their centroid distances (Figures 4E and S4). This analysis revealed complex biphasic and compensatory interaction patterns during infection. Functional analysis showed that these clusters were predominantly associated with mitochondrial processes, with most identified interactors belonging to clusters 2 and 3. Cluster 2 exhibited a depletion phase (0–6 h), followed by stabilization, and was enriched for TCA cycle enzymes, suggesting that ORF9B may initially target mitochondrial oxidative metabolism to redirect carbon flux toward viral biosynthetic needs. In contrast, cluster 3 displayed progressive recruitment before abrupt depletion at 24 h, with significant enrichment for oxidative phosphorylation components and metabolic precursor-generating enzymes, indicating that ORF9B exploits energy production for viral particle assembly before inducing late-stage host shutoff. These findings

Figure 2. APPLE-MS to identify the ORF9B interactome

(A) APPLE-MS workflow. Following Pupylation of the prey protein, SA-Pup^{E}-prey protein complexes are enriched using biotin agarose under stringent washing conditions, e.g., 8 M urea. Protein interactions are validated by SDS-PAGE or identified by label-free quantitative MS.

(B) Subcellular localization of ORF9B. Confocal microscopy shows co-localization (yellow) of TOMM70 (green) and ORF9B-StrepTagII (red) in HEK293T cells. Nuclei were counterstained with DAPI (blue). Scale bars, 10 μm (representative of n = 3). Normalized fluorescence intensity profiles along the spatial axis (distance) are marked on the right. Shown is quantification of Pearson's correlation coefficient from three independent experiments.

(C) Immunoblot analysis of the APPLE assay with HEK293T cell lysates co-expressing TOMM70 and ORF9B-Twin-Strep tag.

(D and E) ORF9B interactome profiling by APPLE-MS. Shown is a volcano plot of ORF9B interactome (n = 3) identified by (D) APPLE-MS (138 significant hits) and (E) AP-MS (135 significant hits). Differential proteins were identified by two-sided moderated t tests in Perseus (v.2.0). Missing values were imputed using Perseus default settings (normal distribution, width = 0.3, downshift = 1.8). Significant interactors (blue) were defined by false discovery rate (FDR)-adjusted $p < 0.05$ and $\log_2 FC \geq 1.5$ (FC: fold change), while reported interactors in BioGRID are shown in red. Gray points indicate non-significant proteins.

(F) Method comparison. Shown is a Venn diagram of PPIs identified by AP-MS (blue), APPLE-MS (green), and literature-curated ORF9B interactors from BioGRID (purple). Database interactions were filtered for ≥2 supporting publications (BioGRID v.4.4.244). Circle areas are proportional to total identifications.

(G) Schematic of ORF9B protein complexes, showing the primary interactor (light green; TOMM70) and secondary interactors (dark green; direct binding partners of TOMM70) identified by AP-MS and APPLE-MS (BioGRID v.4.4.244).

See also Tables S1 and S2.

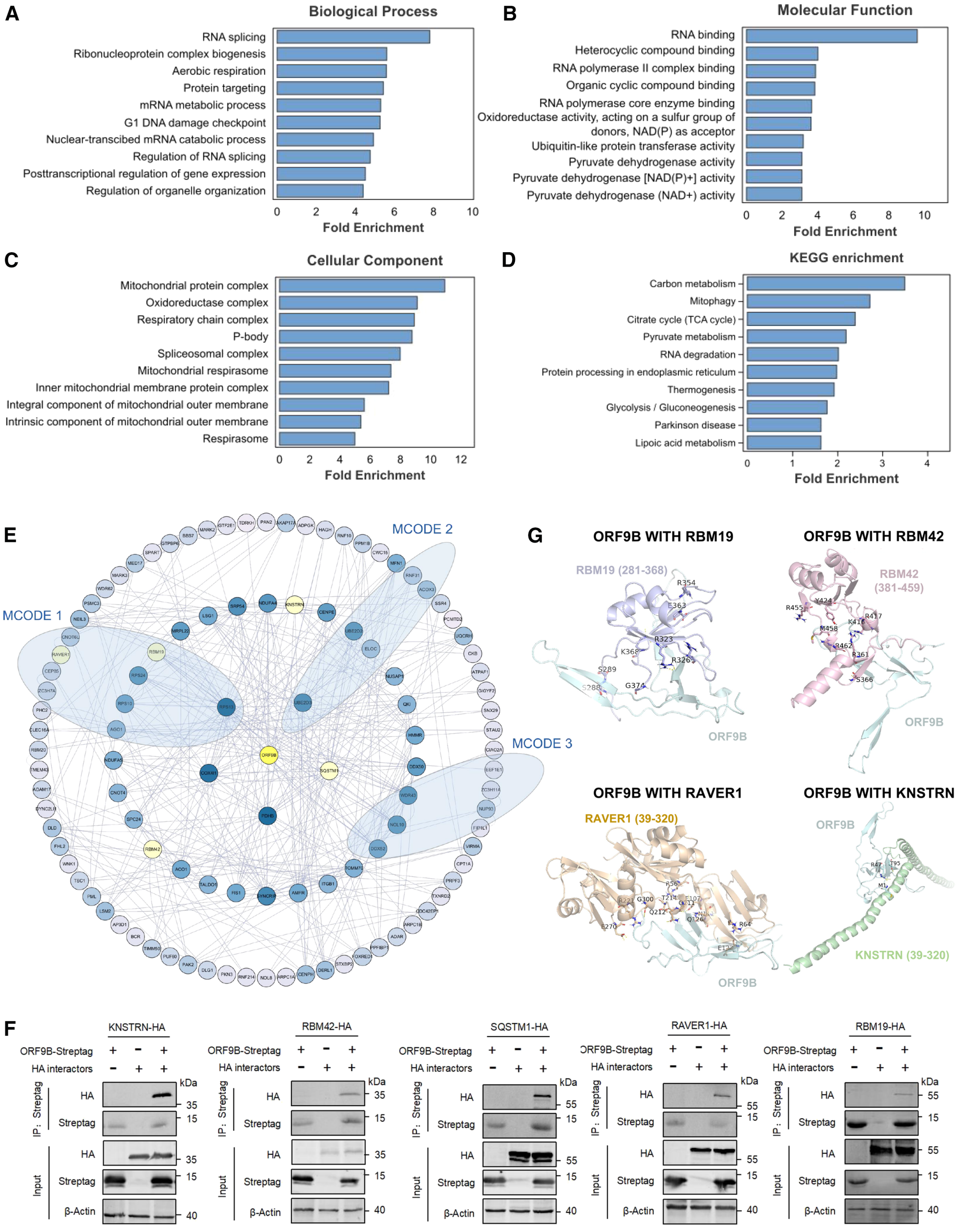

Figure 3. Comprehensive characterization of the ORF9B interactome

(A–C) Functional enrichment analysis. Shown are significantly enriched (FDR < 0.05, hypergeometric test) (A) biological processes, (B) molecular functions, and (C) subcellular localization among ORF9B interactors ($n = 3$). Bar length represents $-\log_{10}$(FDR).

(D) Pathway analysis. Shown is KEGG pathway enrichment of ORF9B interactors ($n = 3$). The bar plot displays significantly enriched KEGG pathways (FDR < 0.05, hypergeometric test) among ORF9B-associated proteins identified by APPLE-MS.

(E) ORF9B interaction network topology. Shown is Cytoscape visualization of ORF9B-associated proteins (nodes) and their interactions (edges). Node color intensity scales with degree (range: 1–25), highlighting proteins for subsequent verification. Three functional modules were identified by MCODE (a Cytoscape plugin) with parameters (degree cutoff $\geq$ 5; node score cutoff $\geq$ 0.4; K-core $\geq$ 2; maximum depth: 100). Module 1, SARS-CoV-1 modulates host translation

(legend continued on next page)

suggest that ORF9B hijacks mitochondrial functions in a temporally coordinated manner. In the early phase of infection, ORF9B disrupts the TCA cycle to accumulate intermediates for viral replication. As infection progresses (6–18 h), ORF9B targets oxidative phosphorylation complexes, particularly components of the electron transport chain, to redirect host energy production toward viral needs. In the late infection phase (>18 h), ORF9B orchestrates a host metabolic shutdown, potentially via inhibition of mitochondrial import machinery or activation of proteolytic pathways. This shutdown may serve dual purposes: suppressing antiviral signaling pathways while facilitating viral particle release. Additionally, we observed that ORF9B modulates mitochondrial gene expression (cluster 1).

In summary, our study establishes the first time-resolved interactome of ORF9B, uncovering novel binding partners and demonstrating its stage-dependent regulation of host mitochondrial processes during poly(I:C)-induced antiviral responses. These results provide crucial insights into how SARS-CoV-2 reprograms host metabolism through ORF9B's phase-specific protein interactions, identifying potential targets for interrupting viral replication cycles.

APPLE-MS for studying endogenous PPIs

In endogenous PPI studies, using small affinity tags such as Twin-Strep (~2–3 kDa) has notable advantages compared to employing larger PL enzymes (~20–50 kDa). Their compact size simplifies genetic insertion and minimizes potential interference with the protein's native function. These features make methods like APPLE-MS particularly suitable for mapping PPIs under near-physiological conditions. PIN1, a phosphorylation-specific prolyl isomerase that catalyzes the *cis-trans* isomerization of phosphorylated Ser/Thr-Pro motifs, serves as a critical regulator of cell cycle progression, transcriptional control, and neurodegenerative pathways.[24] To comprehensively characterize the PIN1 interactome under near-physiological expression conditions, we used CRISPR-Cas9 to insert a Twin-Strep tag at the native PIN1 locus, facilitating extensive PPI mapping via APPLE-MS at endogenous expression levels (Figures 5A–5C). Comparative analyses clearly demonstrated the superior performance of APPLE-MS over conventional AP-MS (Figures 5D and 5E), revealing significantly more known PIN1 interactors, including low-abundance regulators such as ITGA4, with improved signal-to-noise ratios and greater enrichment (Figures 5F and 5G; Table S4). These findings underscore APPLE-MS's enhanced sensitivity for delineating endogenous protein networks under native physiological conditions.

GO enrichment analysis (Figures 5H and S5) of the APPLE-MS-derived PIN1 interactome identified two functionally significant pathways beyond its canonical roles: ncRNA processing and DNA replication machinery. Enrichment of DNA replication components (e.g., RFC1 and the MCM complex) aligns with prior reports indicating PIN1's S phase-specific nuclear localization[25] and its role in replication fork stabilization.[26] These results reinforce PIN1's function as a guardian of genomic stability, where its prolyl-isomerase activity modulates replication stress responses by facilitating fork protection complexes, coordinating checkpoint signaling during replication stress, and sustaining replisome component activity under genomic insult. Additionally, identifying MNAT highlights PIN1's broader involvement in cell cycle regulation, notably at the G1/S transition (Figure 5I). These discoveries position PIN1's isomerase activity as a spatiotemporal regulator that simultaneously ensures genomic fidelity and fine-tunes gene expression networks, particularly significant in oncogenic and neurodegenerative contexts where both processes are commonly dysregulated.

APPLE-MS to identify membrane interactors

To evaluate our method's capability for *in situ* identification of cell-surface PPIs, we employed the APPLE-MS assay to profile the interactome of glucagon-like peptide 1 (GLP-1), a hormone critically relevant to diabetes and obesity management (Figure 6A). Given GLP-1's pleiotropic effects beyond glycemic control,[27] a detailed characterization of its receptor complexes could identify novel therapeutic targets and mechanisms underlying its extrapancreatic actions. For validation, we established a model system by transiently transfecting HEK293T cells with a GLP-1 receptor (GLP-1R) expression plasmid. Notably, all PL reactions were conducted directly on adherent cells in culture dishes, maintaining native membrane contexts, physiological receptor conformation, and endogenous interaction kinetics for PPI capture. Western blot analysis confirmed SA-Pup^E conjugation to GLP-1R (Figure 6B), and immunofluorescence assays validated GLP-1-Twin-Strep tag/GLP-1R complexes at the plasma membrane (Figure 6C), confirming efficient labeling capability and interaction specificity. Subsequently, MS effectively identified membrane interactors, including GLP-1R, in overexpressing cells, affirming APPLE-MS's sensitivity for membrane proteins (Figure 6D; Table S5). Our analysis also detected multiple GLP-1-associated proteins from the BioGRID database. We propose two plausible explanations for these interactions: either these proteins represent endogenous GLP-1-binding partners unaffected by GLP-1R transfection, or they indirectly associate with GLP-1 through receptor-mediated interactions, possibly forming ternary complexes or participating in GLP-1R-dependent signaling networks, positioning GLP-1R as a central regulator of multiprotein assemblies.

Next, we applied APPLE-MS to INS-1E cells, a well-characterized rodent pancreatic β cell line expressing endogenous GLP-1R and native membrane polarity. Our analysis identified 301 putative interactors, including five previously reported GLP-1R-associated proteins from BioGRID and four indicated in Dang et al.[28] (Figure 6E; Table S6). Notably, our method uncovered unexpected co-receptors and transporter complexes (Figure 6F), demonstrating its potential for revealing novel GPCR signaling

machinery (R-HSA-9735869, FDR = 0.001); module 2, peroxisomal protein import (R-HSA-9033241, FDR = 7.47×10^{-4}); module 3, transport of the mature transcript to the cytoplasm (R-HSA-72202, FDR = 3.70×10^{-4}).

(F) CoIP validation. Shown is Strep tag pull-down of five candidate interactors in HEK293T cells.

(G) Predicted binding modes of ORF9B with candidate interactors. Molecular dynamics simulation (500 ns) reveals the lowest-energy binding conformation of ORF9B (cyan) complexed with candidate proteins (root-mean-square deviation [RMSD] cutoff = 2.0 Å). Key interfacial residues mediate hydrophobic interactions.

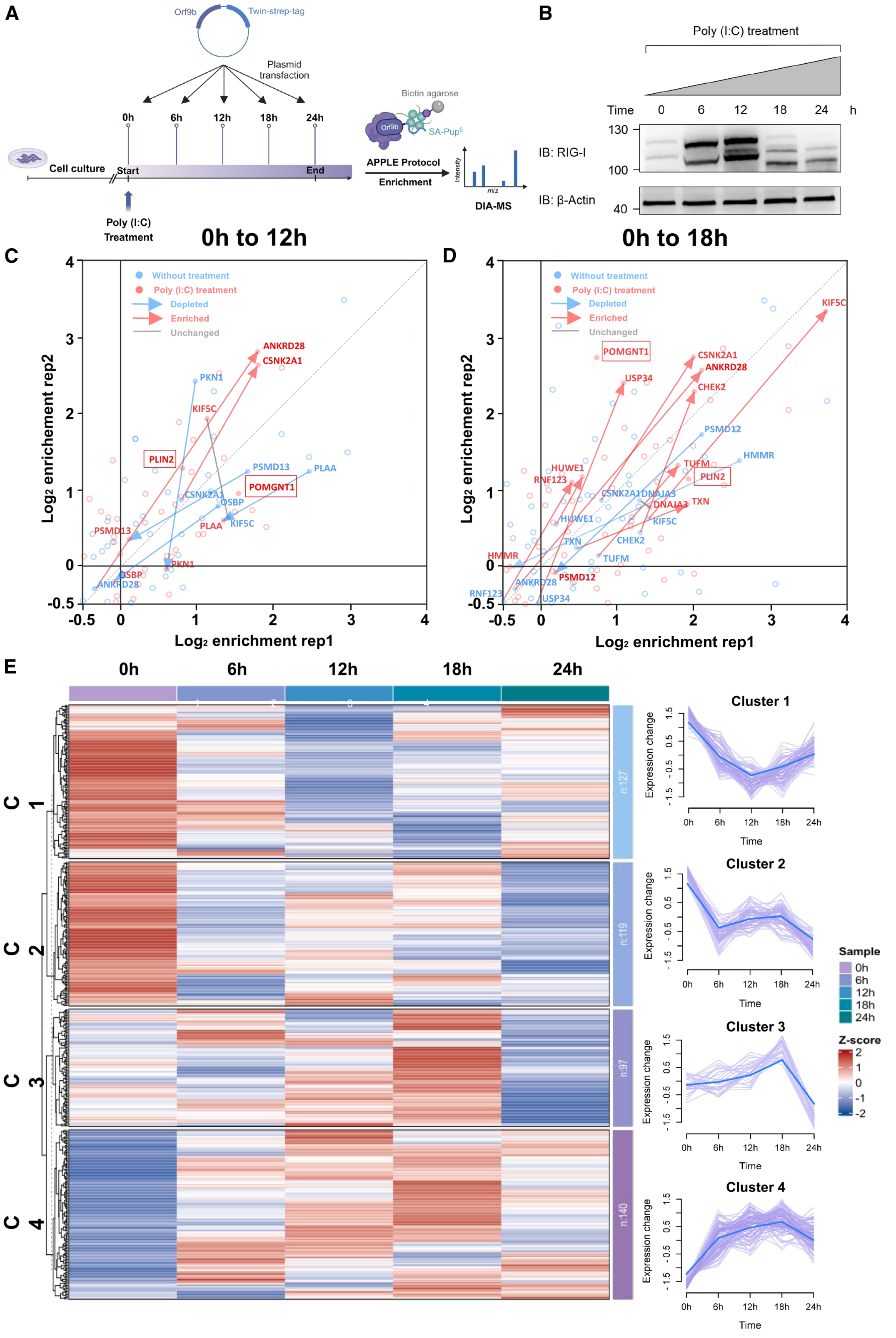

(legend on next page)

CellPress
OPEN ACCESS

network components. The interactome analysis highlighted a complex GLP-1 signaling network featuring functionally significant clusters. Key ATPase components (ATO5MF, BRD8, POLE3, and ATP1A4) and transporter complexes (ABHD6, CLIC5, NDUFS1, NDUFA11, etc.) potentially coordinate ion homeostasis and energy metabolism, with ATP1A4 emerging as a possible molecular bridge between membrane potential regulation and GLP-1-dependent insulin secretion. The detection of receptor-associated proteins such as DNM1 and GLP-1R implies sophisticated regulation of receptor endocytosis and cyclic AMP/PKA signaling kinetics, consistent with GLP-1's established role in glucose-stimulated insulin secretion.[29]

These findings significantly enhance our mechanistic understanding of GLP-1 by revealing its involvement in membrane dynamics and neurometabolic interfaces. The APPLE-MS approach, leveraging PL, robustly captured context-specific interactors, providing an effective platform for future studies of GLP-1's tissue-specific roles. Notably, while our current work focused on the steady-state interactome, the APPLE-MS platform enables future time-resolved studies of GLP-1R or other membrane receptors through controlled addition of Twin-Strep-tagged ligands. This methodological advancement facilitates systematic exploration of ligand-dependent receptor interactome dynamics in native cellular environments.

DISCUSSION

In this study, we introduced APPLE-MS and demonstrated its effectiveness in revealing PPIs that are elusive to traditional methodologies. APPLE-MS synergizes the complementary strengths of AP-MS and PL, enabling detection of a more complete interactome for a given bait protein. By combining the high specificity of Twin-Strep tag enrichment with the proximity-dependent labeling capability of PafA, APPLE-MS effectively captures weak, transient, and membrane-associated interactions in their native cellular context. Systematic comparisons with conventional AP-MS and PL techniques(e.g., BioID, TurboID, and APEX) (Table 1) highlight the contextual utility of APPLE-MS. For instance, while TurboID excels in temporal studies,[30] and APEX is unparalleled for organelle-specific mapping,[31,32] APPLE-MS demonstrates strengths in preserving transient interactions through covalent Pup^E labeling under stringent wash conditions while minimizing bait perturbation via its small Twin-Strep tag. These technical features contribute to enhanced detection of transient interactions, improved signal-to-noise ratios, and more comprehensive interactome coverage relative to standard AP-MS approaches. Notably, APPLE-MS maintains compatibility with endogenous protein expression levels, as evidenced by our CRISPR-mediated tagging of PIN1. This endogenous tagging strategy helps preserve physiological protein interaction networks while reducing potential artifacts associated with overexpression systems. Nevertheless, we stress that APPLE-MS should be viewed as a complementary rather than replacement technology. It is particularly suited for studying labile interactions or membrane-associated complexes where conventional methods face limitations.

This work builds upon our previous work,[40] development of SPIDER (specific Pupylation as identity reporter), which utilized PafA-mediated PupE labeling coupled with the streptavidin-biotin system to efficiently capture protein interactions with diverse biomolecules (e.g., nucleic acids and small molecules), including m^6A-binding proteins and SARS-CoV-2 membrane receptors. While SPIDER excelled in *in vitro* applications, its reliance on biotinylation limited its utility for *in vivo* interactome profiling. By replacing biotin with the Twin-Strep tag, APPLE-MS overcomes this constraint, enabling *in situ* labeling of endogenous protein complexes while retaining the covalent linkage advantages of Pup^E.

Using the well-characterized SPOP MATH domain-peptide interaction model, we established that APPLE-MS can reliably detect interactions with K_D values of up to 76 μM. This sensitivity significantly surpasses conventional AP-MS, which typically fails to identify interactions weaker than ~10 μM due to the noncovalent nature that could not endure stringent washing steps.[41] The covalent labeling mechanism of PafA ensures the stable capture of even low-abundance or transient interactors, addressing a key limitation of non-covalent approaches. Direct comparisons with AP-MS further validated APPLE-MS's enhanced capability, as exemplified by a 4.07-fold increase in high-confidence interactors for SARS-CoV-2 ORF9B with reduced non-specific background. Additionally, APPLE-MS exhibited superior performance in detecting secondary complex

Figure 4. APPLE-MS to identify the dynamic interactome of ORF9B

(A) Workflow of time-course interactome proteomic analysis at five poly(I:C) stimulation time points (0, 6, 12, 18, and 24 h), with three independent biological replicates collected for each time point.

(B) Western blot analysis showing poly(I:C)-induced RIG-I expression dynamics.

(C and D) Dynamic changes in the ORF9B interactome following poly(I:C) stimulation at 12 h (C) and 18 h (D) relative to baseline (0 h). Scatterplot analysis comparing $\log_2$ (fold change) enrichment of ORF9B interactors between untreated (0 h) and poly(I:C)-treated (12 or 18 h, 1 μg/mL) conditions demonstrates stimulus-dependent interaction changes, with each point representing protein enrichment in duplicate experiments (*x*/*y* axes: replicate 1/replicate 2 $\log_2$FC values). Red points represent poly(I:C)-treated samples ($n = 3$ biological replicates), whereas blue points denote untreated controls. Red arrows highlight proteins with increased association, whereas blue arrows mark those with decreased binding ($\log_2FC \leq -1.5$, FDR < 0.05). Gray lines indicate proteins showing no significant change ($|\log_2FC| < 1$). Statistical thresholds were established by two-tailed moderated t test (Benjamini-Hochberg adjustment), with dashed diagonal lines marking x=y reference.

(E) Temporal clustering of the ORF9B interactome during poly(I:C) transfection.

Shown is hierarchical clustering (left) of $\log_2$FC values for known (BioGRID) and novel interactors identified in this study, with proteins grouped by mfuzz time-course clustering. The optimal number of clusters (k = 4, m = 1.25) was determined by (1) minimum centroid distance (>0.8) to ensure distinct expression trajectories and (2) the knee point in the sum of squares error (SSE) curve (Figure S4), where additional clusters no longer significantly reduced variance. Right: Mfuzz time-course clustering of interaction trajectories, with thick blue lines representing cluster centroids. Each line represents an individual protein.

See also Table S3.

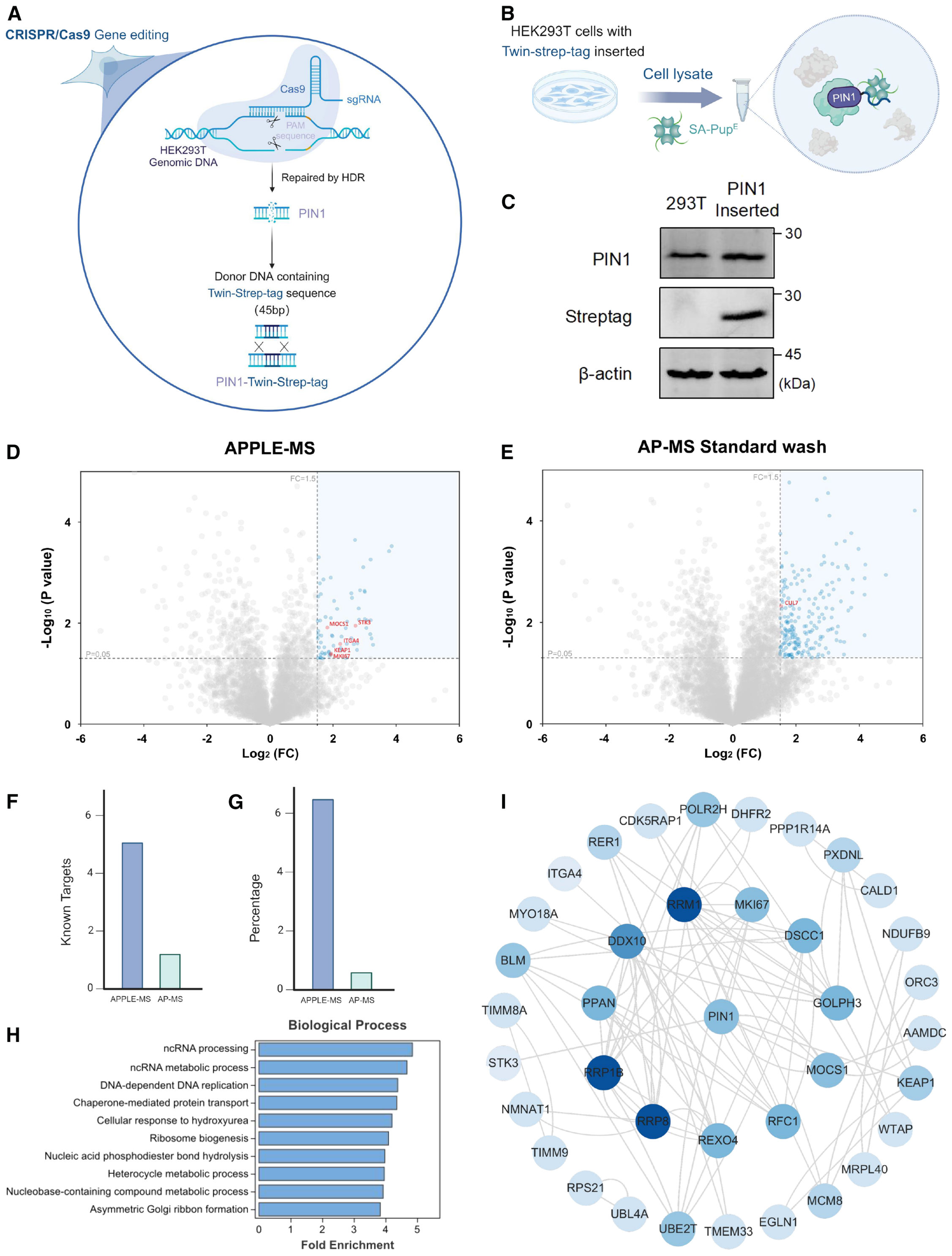

Figure 5. APPLE-MS to study endogenous proteins

(A) Genome engineering strategy. Shown is homology-directed repair (HDR)-mediated C-terminal Twin-Strep tag insertion at the PIN1 locus.

(B) Endogenous PIN1 interaction profiling by APPLE-MS.

(C) Tag validation by western blot confirming Twin-Strep tag insertion.

(D and E) PIN1 interactome profiling. Shown is a volcano plot of PIN1 interactome (n = 3) identified by (D) APPLE-MS (78 significant hits) and (E) AP-MS (200 significant hits). Differential proteins were identified by two-sided moderated t tests in Perseus (v.2.0). Missing values were imputed using Perseus default settings

(legend continued on next page)

CellPress
OPEN ACCESS

components, such as those involved in RNA processing, thus providing deeper insights into complex protein networks. Moreover, APPLE-MS enables the *in situ* labeling of cell-surface proteins, such as GLP-1R, without the need for cell lysis, preserving physiologically relevant membrane contexts and interactions. This is particularly advantageous for studying membrane receptors, where interactions are often dependent on native structural integrity.

Our interactome analyses provided new biological insights across diverse targets. For SARS-CoV-2 ORF9B, APPLE-MS revealed multifaceted roles in viral pathogenesis, including mitochondrial dysfunction, immune evasion, and RNA processing manipulation. Dynamic interactions with key metabolic enzymes and immune regulators, such as RNF123 and TUFM, highlighted ORF9B's complex strategy in reshaping host cellular processes during infection. Similarly, applying APPLE-MS to endogenous PIN1 uncovered involvement in DNA replication stress responses and non-canonical RNA processing pathways. The identified associations, including the MCM complex and RFC1, underscore PIN1's broader regulatory roles beyond its canonical prolyl-isomerase function. For GLP-1R, APPLE-MS identified canonical and previously unexplored co-receptors and signaling components, revealing new potential regulatory mechanisms and therapeutic targets for diabetes and obesity.

Overall, APPLE-MS bridges critical gaps in interactome mapping by integrating the complementary strengths of AP-MS and PL methods. It is broadly applicable to intracellular, membrane, and organellar interactomes across various biological contexts. Future developments, including multiplexed labeling strategies, adaptations for primary cells and tissues, and integration with structural proteomics methods such as cryoelectron microscopy (cryo-EM), promise to further enhance the method's utility and impact.

Limitations of the study

While APPLE-MS offers significant advantages in capturing weak, transient, and membrane-associated interactions, several limitations should be acknowledged. Non-specific labeling, although significantly minimized by optimized reaction conditions, can still introduce background noise. Strategies such as stable-isotope labeling by amino acids in cell culture (SILAC) or tandem mass tag (TMT) labeling could further enhance the specificity by enabling stringent comparative analyses. Additionally, CRISPR-based endogenous tagging, despite providing physiological relevance, involves challenges such as extensive clonal validation and potential off-target genomic effects. To properly position APPLE-MS in the current landscape of PL approaches, future work should include direct comparisons with APEX, TurboID, and related methods.

RESOURCE AVAILABILITY

Lead contact

Requests for further information, resources, and reagents should be directed to and will be fulfilled by the lead contact, Hewei Jiang (jianghewei@lglab.ac.cn).

Materials availability

All unique plasmids and/or reagents generated in the study are available from the lead contact with a completed materials transfer agreement.

Data and code availability

- Original western blot images and microscopy data reported in this paper will be shared by the lead contact upon request.
- The MS proteomics data have been deposited to the ProteomeXchange Consortium via the PRIDE partner repository with the dataset identifier PXD063145.
- This paper does not report original code.
- Any additional information required to reanalyze the data reported in this paper is available from the lead contact upon request.

ACKNOWLEDGMENTS

We thank Hainan Zhang (Institute of Neuroscience, Chinese Academy of Sciences) for technical assistance. This work was funded by Lingang Laboratory (startup fund) and partially supported by the Fourteenth Five-Year National Key Research and Development Program of China (2023YFC2307200), the Natural Science Foundation of China (92374110 and 32271492), the R&D Program of Guangzhou National Laboratory (GZNL2023A01005), and the Shanghai Jiao Tong University Medical-Engineering Interdisciplinary Research Fund (2023-2025).

AUTHOR CONTRIBUTIONS

Conceptualization, H.J. and S.T.; methodology/investigation, S.L., L.X., L.Y., Z.H., L.W., and Y.W.; writing, S.L. and H.J.; resources, Z.H. and S.G.

DECLARATION OF INTERESTS

The authors declare no competing interests.

STAR★METHODS

Detailed methods are provided in the online version of this paper and include the following:

- KEY RESOURCES TABLE
- EXPERIMENTAL MODEL AND STUDY PARTICIPANT DETAILS
  - Cell culture
- METHOD DETAILS
  - Plasmids construction and cloning
  - Protein expression and purification
  - Cell transfection and lysate preparation
  - APPLE-MS assay
  - Immunoblotting
  - Immunofluorescence
  - Affinity purification for protein-protein interactions

(normal distribution, width = 0.3, downshift = 1.8). Significant interactors (blue) were defined by FDR-adjusted $p < 0.05$ and $\log_2$FC ≥ 1.5, while reported interactors in BioGRID are shown in red. Gray points indicate non-significant proteins.
(F and G) Method comparison. Shown are (F) absolute counts and (G) detection rates of known PIN1 interactors.
(H) Functional enrichment. The bar plot displays significantly enriched biological processes (FDR < 0.05, hypergeometric test) among PIN1 interactors identified by APPLE-MS ($n = 3$). The length of each bar corresponds to the $-\log_{10}$(FDR) value, representing the statistical significance of enrichment.
(I) PIN1 interaction network topology. Shown is Cytoscape visualization of PIN1-associated proteins (nodes) and their interactions (edges). Node color intensity scales with degree (range: 1–25), highlighting proteins for subsequent verification.
See also Table S4.

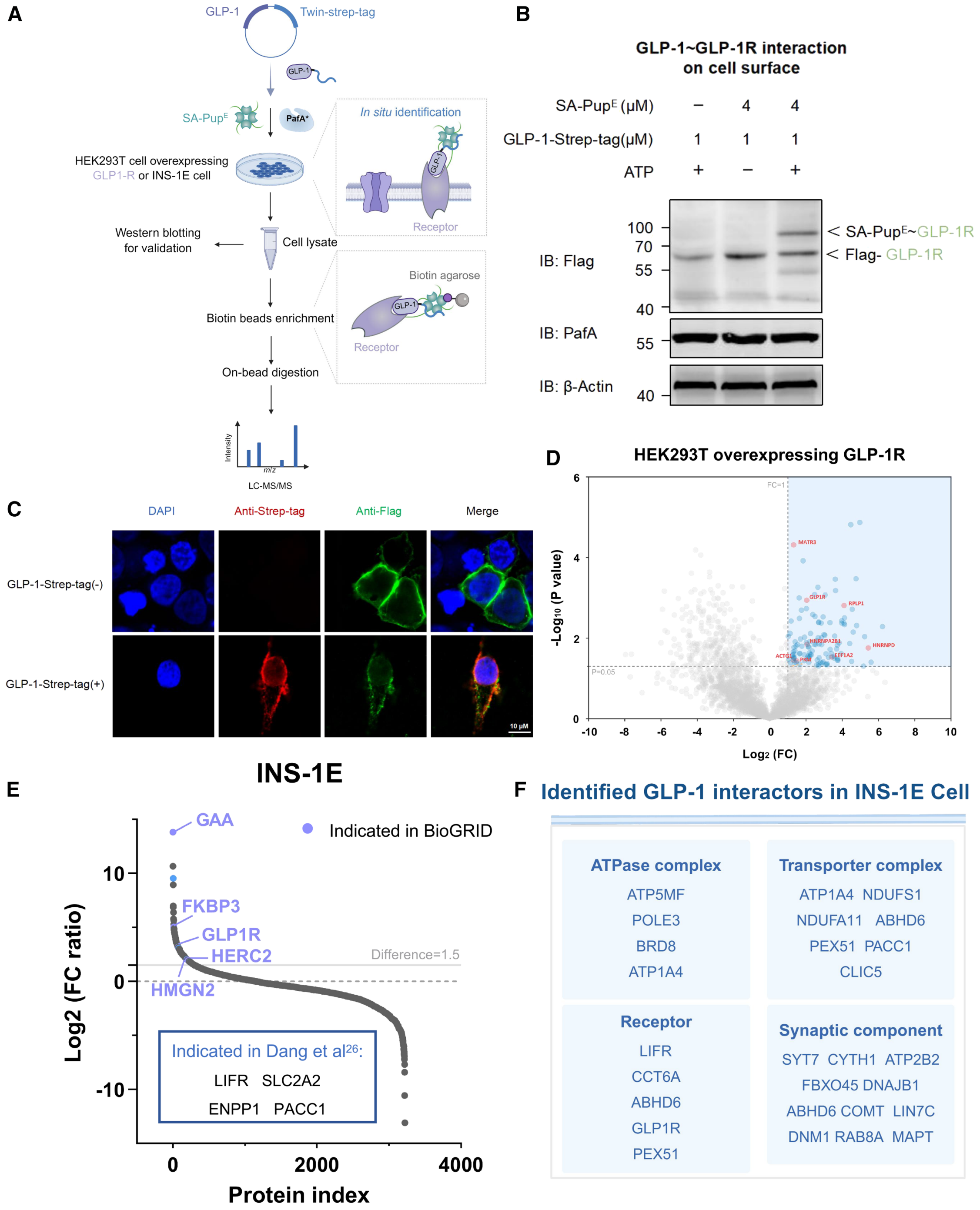

Figure 6. APPLE-MS to identify membrane interactors

(A) Schematic design of APPLE-MS for identifying cell-surface protein interactors. The labeling process is performed *in situ* by removing the culture medium from the dish. Interactions between the Twin-Strep-tagged bait and prey protein are then detected via gel electrophoresis or MS.

(B) Western blot analysis of *in situ* GLP-1-GLP-1R interaction in HEK293T cells using the APPLE assay.

(C) Immunofluorescence imaging demonstrating cell surface colocalization (yellow) of GLP-1R (green) with GLP-1-Twin-Strep tag (red) in HEK293 cells expressing GLP-1R. Anti-Strep tags were used to stain GLP-1-Twin-Strep tag. Nuclei were counterstained with DAPI (blue). Representative images from three independent experiments are shown. Scale bars, 10 μm.

(D) GLP-1 interactome profiling by APPLE-MS. Shown is a volcano plot of the GLP-1 interactome (96 significant hits). Differential proteins were identified by two-sided moderated t tests in Perseus (v.2.0). Missing values were imputed using Perseus default settings (normal distribution, width = 0.3, downshift = 1.8).

(legend continued on next page)

Table 1. Comparison of traditional AP-MS, BioID/TurboID/APEX, and APPLE-MS

Method	Traditional AP-MS	BioID/TurboID/APEX	APPLE-MS
The size of the bait tag	• small (FLAG/HA, 8–15 aa, or GFP fusion ~27 kDa)<br>• minimal functional interference	• large (BirA ~35 kDa, TurboID ~28 kDa, APEX ~27 kDa)<br>• potential interference with bait function (Proc et al.[33])	• small (Twin-Strep tag, 30 aa, ~3 kDa); no large enzyme fusion<br>• minimal functional interference
Technical accessibility	• simple workflow: express the bait-small tag fusion, lyse cells, and perform AP	• more steps but can be performed in live cells<br>• requires bait-enzyme fusion and intracellular biotin supplementation for labeling (BioID & TurboID) (May et al.[34]);	• simple workflow similar to AP: express the bait-small tag fusion with universal PafA enzyme for labeling<br>• no special reagents required
Generality	• functional in almost all human protein expression systems (Goudreault et al.,[35] Bruderer et al.[36])<br>• requires tag or antibody for each bait	• compatible with most cell types<br>• dependent on enzyme compatibility and precise localization	• broad applicability: suitable for cells supporting endogenous CRISPR insertion<br>• universal PafA with modular small tags allows parallel bait processing
Capture of transient/ weak interactions	• limited: weak interactions may dissociate during lysis and washing (Völkel et al.[7])<br>• primarily detects stable complexes	• excellent: covalent labeling captures transient interactions<br>• labeling radius ~tens of nm (May et al.,[34] Nguyen et al.[37]), capturing broad neighboring proteins	• excellent: Pup-mediated covalent linkage captures transient interactions<br>• labeling confined to direct interactors, offering higher specificity
Quantification and reproducibility	• weak interactions show poor reproducibility (Dazard et al.[38]), requiring multiple replicates	• better reproducibility; weak interactions are more consistently detected across replicates<br>• labeling abundance correlates with proximity/frequency	• better reproducibility; stable interactions are consistently detected<br>• quantification via MS intensity, similar to AP-MS but with PL enrichment advantages
Bait construction requirements	• each bait requires a small tag (or specific antibody)<br>• tags are general and easy to clone	• each bait requires fusion to a specific labeling enzyme<br>• Large fusions increase cloning complexity (Qin et al.[39]), necessitating target-specific optimization	• Each bait requires a universal small tag (Twin-Strep-tag);<br>• PafA can be universally provided, eliminating the need for enzyme replacement across different baits

Abbreviations: HA, hemagglutinin; aa, amino acids; nm, nanometers.

  - Poly (I:C) treatment
  - Coimmunoprecipitation
  - Mass spectrometry
  - Proteomics data analysis
  - Generation of endogenous tagging of PIN1 knock-in cell line
  - Structure analysis
- QUANTIFICATION AND STATISTICAL ANALYSIS

SUPPLEMENTAL INFORMATION

Supplemental information can be found online at https://doi.org/10.1016/j.crmeth.2025.101166.

Received: May 27, 2025
Revised: June 25, 2025
Accepted: August 13, 2025
Published: September 8, 2025

REFERENCES

1. Berggård, T., Linse, S., and James, P. (2007). Methods for the detection and analysis of protein–protein interactions. Proteomics *7*, 2833–2842. https://doi.org/10.1002/pmic.200700131.
2. Wodak, S.J., Vlasblom, J., Turinsky, A.L., and Pu, S. (2013). Protein-protein interaction networks: the puzzling riches. Curr. Opin. Struct. Biol. *23*, 941–953. https://doi.org/10.1016/j.sbi.2013.08.002.

Significant interactors (blue) were defined by FDR-adjusted $p < 0.05$ and $\log_2FC \geq 1$, while reported interactors in BioGRID are shown in red. Gray points indicate non-significant proteins ($n = 3$).
(E) Rank plot analysis of $\log_2FC$ ratio values of proteins in two biological replicates. Reported interactors in BioGRID are highlighted in red. Indicated interactors in Dang et al. are highlighted in blue.
(F) Functional categorization. Representative GLP-1R interactors from INS-1E cells are grouped by biological function.
See also Tables S5 and S6.

3. Hayes, S., Malacrida, B., Kiely, M., and Kiely, P.A. (2016). Studying protein-protein interactions: progress, pitfalls and solutions. Biochem. Soc. Trans. *44*, 994–1004. https://doi.org/10.1042/BST20160092.

4. Keskin, O., Tuncbag, N., and Gursoy, A. (2016). Predicting Protein-Protein Interactions from the Molecular to the Proteome Level. Chem. Rev. *116*, 4884–4909. https://doi.org/10.1021/acs.chemrev.5b00683.

5. Durham, J., Zhang, J., Humphreys, I.R., Pei, J., and Cong, Q. (2023). Recent advances in predicting and modeling protein-protein interactions. Trends Biochem. Sci. *48*, 527–538. https://doi.org/10.1016/j.tibs.2023.03.003.

6. Paul, F.E., Hosp, F., and Selbach, M. (2011). Analyzing protein-protein interactions by quantitative mass spectrometry. Methods *54*, 387–395. https://doi.org/10.1016/j.ymeth.2011.03.001.

7. Völkel, P., Le Faou, P., and Angrand, P.O. (2010). Interaction proteomics: characterization of protein complexes using tandem affinity purification-mass spectrometry. Biochem. Soc. Trans. *38*, 883–887. https://doi.org/10.1042/BST0380883.

8. Wolf, P., Gavins, G., Beck-Sickinger, A.G., and Seitz, O. (2021). Strategies for Site-Specific Labeling of Receptor Proteins on the Surfaces of Living Cells by Using Genetically Encoded Peptide Tags. Chembiochem *22*, 1717–1732. https://doi.org/10.1002/cbic.202000797.

9. Yang, Z., Wang, C., Zhou, Q., An, J., Hildebrandt, E., Aleksandrov, L.A., Kappes, J.C., DeLucas, L.J., Riordan, J.R., Urbatsch, I.L., et al. (2014). Membrane protein stability can be compromised by detergent interactions with the extramembranous soluble domains. Protein Sci. *23*, 769–789. https://doi.org/10.1002/pro.2460.

10. Sharifi Tabar, M., Parsania, C., Chen, H., Su, X.D., Bailey, C.G., and Rasko, J.E.J. (2022). Illuminating the dark protein-protein interactome. Cell Rep. Methods *2*, 100275. https://doi.org/10.1016/j.crmeth.2022.100275.

11. Liu, X., Salokas, K., Weldatsadik, R.G., Gawriyski, L., and Varjosalo, M. (2020). Combined proximity labeling and affinity purification-mass spectrometry workflow for mapping and visualizing protein interaction networks. Nat. Protoc. *15*, 3182–3211. https://doi.org/10.1038/s41596-020-0365-x.

12. Guth, E., Thommen, M., and Weber-Ban, E. (2011). Mycobacterial ubiquitin-like protein ligase PafA follows a two-step reaction pathway with a phosphorylated pup intermediate. J. Biol. Chem. *286*, 4412–4419. https://doi.org/10.1074/jbc.M110.189282.

13. Junttila, M.R., Saarinen, S., Schmidt, T., Kast, J., and Westermarck, J. (2005). Single-step Strep-tag purification for the isolation and identification of protein complexes from mammalian cells. Proteomics *5*, 1199–1203. https://doi.org/10.1002/pmic.200400991.

14. Zhuang, M., Calabrese, M.F., Liu, J., Waddell, M.B., Nourse, A., Hammel, M., Miller, D.J., Walden, H., Duda, D.M., Seyedin, S.N., et al. (2009). Structures of SPOP-substrate complexes: insights into molecular architectures of BTB-Cul3 ubiquitin ligases. Mol. Cell *36*, 39–50. https://doi.org/10.1016/j.molcel.2009.09.022.

15. Han, L., Zhuang, M.W., Deng, J., Zheng, Y., Zhang, J., Nan, M.L., Zhang, X.J., Gao, C., and Wang, P.H. (2021). SARS-CoV-2 ORF9b antagonizes type I and III interferons by targeting multiple components of the RIG-I/MDA-5-MAVS, TLR3-TRIF, and cGAS-STING signaling pathways. J. Med. Virol. *93*, 5376–5389. https://doi.org/10.1002/jmv.27050.

16. Jiang, H.W., Zhang, H.N., Meng, Q.F., Xie, J., Li, Y., Chen, H., Zheng, Y.X., Wang, X.N., Qi, H., Zhang, J., et al. (2020). SARS-CoV-2 Orf9b suppresses type I interferon responses by targeting TOM70. Cell. Mol. Immunol. *17*, 998–1000. https://doi.org/10.1038/s41423-020-0514-8.

17. Mellacheruvu, D., Wright, Z., Couzens, A.L., Lambert, J.P., St-Denis, N.A., Li, T., Miteva, Y.V., Hauri, S., Sardiu, M.E., Low, T.Y., et al. (2013). The CRAPome: a contaminant repository for affinity purification-mass spectrometry data. Nat. Methods *10*, 730–736. https://doi.org/10.1038/nmeth.2557.

18. Gao, X., Zhu, K., Qin, B., Olieric, V., Wang, M., and Cui, S. (2021). Crystal structure of SARS-CoV-2 Orf9b in complex with human TOM70 suggests unusual virus-host interactions. Nat. Commun. *12*, 2843. https://doi.org/10.1038/s41467-021-23118-8.

19. Ben-Oz, B.M., Machour, F.E., Nicola, M., Argoetti, A., Polyak, G., Hanna, R., Kleifeld, O., Mandel-Gutfreund, Y., and Ayoub, N. (2023). A dual role of RBM42 in modulating splicing and translation of CDKN1A/p21 during DNA damage response. Nat. Commun. *14*, 7628. https://doi.org/10.1038/s41467-023-43495-6.

20. Zhang, J., Tripathi, D.N., Jing, J., Alexander, A., Kim, J., Powell, R.T., Dere, R., Tait-Mulder, J., Lee, J.H., Paull, T.T., et al. (2015). ATM functions at the peroxisome to induce pexophagy in response to ROS. Nat. Cell Biol. *17*, 1259–1269. https://doi.org/10.1038/ncb3230.

21. Gromak, N., Rideau, A., Southby, J., Scadden, A.D.J., Gooding, C., Hüttelmaier, S., Singer, R.H., and Smith, C.W.J. (2003). The PTB interacting protein raver1 regulates alpha-tropomyosin alternative splicing. EMBO J. *22*, 6356–6364. https://doi.org/10.1093/emboj/cdg609.

22. Lever, A.R., Park, H., Mulhern, T.J., Jackson, G.R., Comolli, J.C., Borenstein, J.T., Hayden, P.J., and Prantil-Baun, R. (2015). Comprehensive evaluation of poly(I:C) induced inflammatory response in an airway epithelial model. Physiol. Rep. *3*, e12334. https://doi.org/10.14814/phy2.12334.

23. Kumar, L., and E Futschik, M. (2007). Mfuzz: a software package for soft clustering of microarray data. Bioinformation *2*, 5–7. https://doi.org/10.6026/97320630002005.

24. Maggio, J., Armando, R., Balcone, L., Vilarullo, R., Casco, M., Mengual Gomez, D., and Gomez, D. (2023). Role of PIN1 in human pathology: Cellular regulation, pathogenesis and therapeutic implications. World Acad. Sci. J. *6*, 5. https://doi.org/10.3892/wasj.2023.220.

25. Cheng, C.W., and Tse, E. (2018). PIN1 in Cell Cycle Control and Cancer. Front. Pharmacol. *9*, 1367. https://doi.org/10.3389/fphar.2018.01367.

26. Daza-Martin, M., Starowicz, K., Jamshad, M., Tye, S., Ronson, G.E., MacKay, H.L., Chauhan, A.S., Walker, A.K., Stone, H.R., Beesley, J.F.J., et al. (2019). Isomerization of BRCA1-BARD1 promotes replication fork protection. Nature *571*, 521–527. https://doi.org/10.1038/s41586-019-1363-4.

27. Al-Noshokaty, T.M., Abdelhamid, R., Abdelmaksoud, N.M., Khaled, A., Hossam, M., Ahmed, R., Saber, T., Khaled, S., Elshaer, S.S., and Abulsoud, A.I. (2025). Unlocking the multifaceted roles of GLP-1: Physiological functions and therapeutic potential. Toxicol. Rep. *14*, 101895. https://doi.org/10.1016/j.toxrep.2025.101895.

28. Dang, T., Yu, J., Cao, Z., Zhang, B., Li, S., Xin, Y., Yang, L., Lou, R., Zhuang, M., and Shui, W. (2025). Endogenous cell membrane interactome mapping for the GLP-1 receptor in different cell types. Nat. Chem. Biol. *21*, 256–267. https://doi.org/10.1038/s41589-024-01714-1.

29. Tomas, A., Jones, B., and Leech, C. (2020). New Insights into Beta-Cell GLP-1 Receptor and cAMP Signaling. J. Mol. Biol. *432*, 1347–1366. https://doi.org/10.1016/j.jmb.2019.08.009.

30. Kim, H.B., and Kim, K.E. (2024). Precision proteomics with TurboID: mapping the suborganelle landscape. Korean J. Physiol. Pharmacol. *28*, 495–501. https://doi.org/10.4196/kjpp.2024.28.6.495.

31. Zhu, H., Tamura, T., and Hamachi, I. (2019). Chemical proteomics for subcellular proteome analysis. Curr. Opin. Chem. Biol. *48*, 1–7. https://doi.org/10.1016/j.cbpa.2018.08.001.

32. Lee, S.Y., Kang, M.G., Park, J.S., Lee, G., Ting, A.Y., and Rhee, H.W. (2016). APEX Fingerprinting Reveals the Subcellular Localization of Proteins of Interest. Cell Rep. *15*, 1837–1847. https://doi.org/10.1016/j.celrep.2016.04.064.

33. Proc, J.L., Kuzyk, M.A., Hardie, D.B., Yang, J., Smith, D.S., Jackson, A.M., Parker, C.E., and Borchers, C.H. (2010). A quantitative study of the effects of chaotropic agents, surfactants, and solvents on the digestion efficiency of human plasma proteins by trypsin. J. Proteome Res. *9*, 5422–5437. https://doi.org/10.1021/pr100656u.

34. May, D.G., Scott, K.L., Campos, A.R., and Roux, K.J. (2020). Comparative Application of BioID and TurboID for Protein-Proximity Biotinylation. Cells *9*, 1070. https://doi.org/10.3390/cells9051070.

35. Goudreault, M., D'Ambrosio, L.M., Kean, M.J., Mullin, M.J., Larsen, B.G., Sanchez, A., Chaudhry, S., Chen, G.I., Sicheri, F., Nesvizhskii, A.I., et al. (2009). A PP2A phosphatase high density interaction network identifies a novel striatin-interacting phosphatase and kinase complex linked to the cerebral cavernous malformation 3 (CCM3) protein. Mol. Cell. Proteomics *8*, 157–171. https://doi.org/10.1074/mcp.M800266-MCP200.

36. Bruderer, R., Bernhardt, O.M., Gandhi, T., Miladinović, S.M., Cheng, L.Y., Messner, S., Ehrenberger, T., Zanotelli, V., Butscheid, Y., Escher, C., et al. (2015). Extending the limits of quantitative proteome profiling with data-independent acquisition and application to acetaminophen-treated three-dimensional liver microtissues. Mol. Cell. Proteomics *14*, 1400–1410. https://doi.org/10.1074/mcp.M114.044305.

37. Nguyen, T.M.T., Kim, J., Doan, T.T., Lee, M.W., and Lee, M. (2020). APEX Proximity Labeling as a Versatile Tool for Biological Research. Biochemistry *59*, 260–269. https://doi.org/10.1021/acs.biochem.9b00791.

38. Dazard, J.E., Saha, S., and Ewing, R.M. (2012). ROCS: a reproducibility index and confidence score for interaction proteomics studies. BMC Bioinf. *13*, 128. https://doi.org/10.1186/1471-2105-13-128.

39. Qin, W., Cho, K.F., Cavanagh, P.E., and Ting, A.Y. (2021). Deciphering molecular interactions by proximity labeling. Nat. Methods *18*, 133–143. https://doi.org/10.1038/s41592-020-01010-5.

40. Jiang, H.W., Chen, H., Zheng, Y.X., Wang, X.N., Meng, Q., Xie, J., Zhang, J., Zhang, C., Xu, Z.W., Chen, Z.Q., et al. (2023). Specific pupylation as IDEntity reporter (SPIDER) for the identification of protein-biomolecule interactions. Sci. China Life Sci. *66*, 1869–1887. https://doi.org/10.1007/s11427-023-2316-2.

41. Yue, S., Xu, P., Cao, Z., and Zhuang, M. (2022). PUP-IT2 as an alternative strategy for PUP-IT proximity labeling. Front. Mol. Biosci. *9*, 1007720. https://doi.org/10.3389/fmolb.2022.1007720.

42. Schneider, C.A., Rasband, W.S., and Eliceiri, K.W. (2012). NIH Image to ImageJ: 25 years of image analysis. Nat. Methods *9*, 671–675. https://doi.org/10.1038/nmeth.2089.

43. Tyanova, S., Temu, T., Sinitcyn, P., Carlson, A., Hein, M.Y., Geiger, T., Mann, M., and Cox, J. (2016). The Perseus computational platform for comprehensive analysis of (prote)omics data. Nat. Methods *13*, 731–740. https://doi.org/10.1038/nmeth.3901.

44. Shannon, P., Markiel, A., Ozier, O., Baliga, N.S., Wang, J.T., Ramage, D., Amin, N., Schwikowski, B., and Ideker, T. (2003). Cytoscape: a software environment for integrated models of biomolecular interaction networks. Genome Res. *13*, 2498–2504. https://doi.org/10.1101/gr.1239303.

45. Perez-Riverol, Y., Csordas, A., Bai, J., Bernal-Llinares, M., Hewapathirana, S., Kundu, D.J., Inuganti, A., Griss, J., Mayer, G., Eisenacher, M., et al. (2019). The PRIDE database and related tools and resources in 2019: improving support for quantification data. Nucleic Acids Res. *47*, D442–D450. https://doi.org/10.1093/nar/gky1106.

STAR★METHODS

KEY RESOURCES TABLE

REAGENT or RESOURCE	SOURCE	IDENTIFIER
Antibodies		
WB: GFP-Tag(7G9) mAb	Abmart	Cat#M20004; RRID:AB_2619674
WB: DYKDDDDK Tag (D6W5B) Rabbit mAb	Cell Signaling Technology	Cat#14793; RRID:AB_2572291
WB: Mouse anti His-Tag mAb	ABclonal	Cat#AE003; RRID:AB_2728734
WB: β-Actin Mouse mAb	ABclonal	Cat#AC004; RRID:AB_2737399
WB/IF: TOM70 (E7E1M) Rabbit mAb	Cell Signaling Technology	Cat#65619; RRID:AB_3411916
WB: Myc-Tag Rabbit mAb	ABclonal	Cat#AE070; RRID:AB_2863795
WB: Mouse anti HA-Tag mAb	ABclonal	Cat#AE008; RRID:AB_2770404
WB: Goat Anti-Rabbit IgG (H&L) HRP	Abmart	Cat#M212115; RRID:AB_2916063
WB: Goat Anti-Mouse IgG (H&L) HRP	Abmart	Cat#M212108; RRID:AB_2916064
WB: Anti-twin-strep-tag Antibody (SAA0348)	AntibodySystem	Cat#RGK26103;RRID: N/A
WB: RIG-I/DDX58 Rabbit Polyclonal Antibody	Beyotime	Cat#AF7890;RRID:AB: N/A
IF: Goat anti-Mouse IgG, IgM (H + L) Secondary Antibody, Alexa Fluortm 488	Invitrogen	Cat#A28175;RRID:AB_2536161
Bacterial and virus strains		
E. coli DH5α	Sangon Biotech	Cat#B528413-0100
E. coli BL21	Sangon Biotech	Cat#B528414-0100
Chemicals, peptides, and recombinant proteins		
Synthetic peptide 1 (LACDEVTSTTSSSTA)	Synthesized by GL Biochem (Shanghai)	N/A
Polyethyleneimine	Polysciences	Cat#24765-100
IF: Alexa Fluor® 647 Streptavidin	Invitrogen	Cat#S21374
Urea	BBI	Cat#A600148-0500; CAS: [57-13-6]
Tween 20	Greagent	Cat#01222003; CAS: 9005-64-5
Triton X-100	Adamas life	Cat#013516633; CAS: 9002-93-1
DL-dithiothreitol (DTT)	BBI	Cat#A620058-0025; CAS: [3483-12-3]
Tris(hydroxymethyl)aminoethane	Sangon	Cat#A501492-0500; CAS: [77-86-1]
Phenylmethanesulfonyl fluoride (PMSF)	BBI	Cat#A610425-0005 CAS: [329-98-6]
DMEM high glucose	GIBCO	Cat#C11995500BT
Fetal bovine serum (FBS)	BI	Cat#04-001-1ACS
Opti-MEM Reduced Serum Medium	GIBCO	Cat#31985070
Lipofectamine^{TM} 3000 Transfection Reagent	Invitrogen	Cat#L3000015
Critical commercial assays		
BCA assay	Beyotime	Cat#P0009
EndoFree Mini Plasmid Kit	TIANGEN	Cat#DP118-02
Deposited data		
Raw and analyzed mass spectrometry data	This paper	PRIDE:PXD063145
Experimental models: Cell lines		
Human: HEK293T cells	ATCC	CRL-3216
Rat: INS-1E cells	Provided by Shanghai Yaji Biotechnology Co., Ltd	Cat# YS1176C

(*Continued on next page*)

Continued

REAGENT or RESOURCE	SOURCE	IDENTIFIER
Oligonucleotides		
HJP019-F: ctgtgtcccacgggctctgcagactct	This paper	N/A
HJP019-R: agttcaccagaacctggcactcaaccaaggag	This paper	N/A
HJP020-F: atgtcaatgtcggcacccactggtc	This paper	N/A
HJP020-R: AACTGTGGGTGGCT CCAGGCGCTcatcttccctcctgccgca	This paper	N/A
HJP021-F: TGGAGGCGGTGGATC TGGCgcggacgaggagaagctg	This paper	N/A
HJP021-R: acggggagggggatttgtaggcaaac	This paper	N/A
HJP022-F: AGCGCCTGGAGCCACC CACAGTTCGAGAAAGGTGGCGG ATCAGGAGGCGGGAG	This paper	N/A
HJP023-R: CTTTTCAAATTGAGGGTGGGAC CAAGCAGAGCCTCCGCTCCCG CCTCCTGATCCGCCAC	This paper	N/A
HJP024-R: GCCAGATCCACCGCCTCCAGAG CCACCTCCGCCAGAGCCGCC CTTTTCAAATTGAGGGT	This paper	N/A
Software and algorithms		
ImageJ	Schneider et al.[42]	https://imagej.nih.gov/ij/
CRAPome	Mellacheruvu et al.[17]	https://reprint-apms.org/?q=chooseworkflow
Muffz	Kumar et al.[23]	https://bioconductor.org/packages/release/bioc/html/Mfuzz.html
Perseus	Tyanova et al.[43]	https://www.maxquant.org/perseus/
GraphPad Prism (version 9.0.1)	GraphPad software	https://www.graphpad.com/scientific-software/prism/
Cytoscape	Shannon et al.[44]	https://cytoscape.org/
PRIDE partner repository	Perez-Riverol et al.[45]	https://www.ebi.ac.uk/pride/
BioRender	Science Suite Inc.	BioRender Online

EXPERIMENTAL MODEL AND STUDY PARTICIPANT DETAILS

Cell culture

APPLE-MS was tested on HEK 293T and INS-1E cells. HEK293T cells were cultured in DMEM (Thermo Fisher Scientific) and INS-1E cells were cultured in RPMI 1640 (Thermo Fisher Scientific). The culture mediums were supplemented with 10% (v/v) heat-inactivated fetal bovine serum (FBS) and 1% Penicillin-Streptomycin. All cell lines were cultured in 37°C and 5% CO_2. Cell passaging was conducted at 2- 4days intervals. No mycoplasma contamination was detected for all cell lines.

METHOD DETAILS

Plasmids construction and cloning

Protein sequences were downloaded from Addgene. The corresponding DNA sequences were codon-optimized and synthesized by Tsingke Biotech (Shanghai, China), and the synthesized genes were cloned into pET28a or pET23a for prokaryotic expression or pcDNA3.1 for eukaryotic expression. All plasmids were constructed using homologous recombination. Target genes were amplified by PCR (Phanta SE Super-Fidelity DNA Polymerase, Vazyme) with primers containing 15–20 bp overlapping regions. Ligated products were transformed into chemically competent E. coli DH5α or BL21 (Sangon Biotech) plated on LB-agar with appropriate antibiotics, and validated by Sanger sequencing (Tsingke Biotech).

Protein expression and purification

E. coli BL21, carrying the expression plasmid, was cultured in LB medium at 37°C until the A_{600} reached 0.6–0.8. Protein expression was induced with 0.5 mmol L−1 isopropyl-β-D-thiogalactoside (IPTG) at 18°C for 16 h. Cell pellets expressing 6×His-tagged proteins were resuspended in lysis buffer (50 mM Tris-HCl, pH 8.0, 500 mM NaCl, 20 mM imidazole) and lysed using a high-pressure homogenizer (Union-Biotech, China). The lysate was clarified by centrifugation (10,000 × *g*, 20 min, 4°C), and the supernatant was incubated with Ni-NTA Agarose (Qiagen). After binding, the resin was washed with lysis buffer and eluted with an imidazole gradient (300 mM in 50 mM Tris-HCl, pH 8.0, 500 mM NaCl).

Cell transfection and lysate preparation

For transfection, we employed polyethylenimine (PEI, 1 mg/mL, Polysciences) at a ratio of 2 μL PEI per 1 μg plasmid DNA (e.g., 4 μL PEI for 2 μg plasmid). The plasmid DNA was first diluted in 50 μL of serum- and antibiotic-free DMEM medium and mixed gently by pipetting. In parallel, the appropriate amount of PEI was diluted in another 50 μL of serum- and antibiotic-free DMEM, while the corresponding amount of PEI was separately diluted in another 50 μL of serum-free DMEM. Within 5 min of preparation, the plasmid and PEI solutions were combined, thoroughly mixed, and incubated at room temperature for 15 min to allow optimal DNA-PEI complex formation. These complexes were then added dropwise to 293T cells that had reached approximately 85% confluency. Following 48 h of incubation to allow protein expression, transfected cells were processed for either immediate analysis or cell lysis.

For protein extraction, transfected cells were first washed with PBS before lysis with 1 mL of M-PER Reagent (Thermo Fisher Scientific) supplemented with 0.5 mM PMSF per 10 cm culture dish. Complete lysis was achieved by gentle shaking for 20 min at room temperature. The resulting lysate was then clarified by centrifugation at 12,000×g for 10 min to remove cellular debris. The protein-rich supernatant was carefully aliquoted and stored at −80°C to preserve protein integrity for subsequent downstream applications.

APPLE-MS assay

For the APPLE experiment validating the interaction between bait peptides and GFP-MATH, Twin-Strep-tag-fused bait peptides 1–3, GFP-MATH, SA-Pup^E, PafA12KR and ATP were co-incubated at 37°C for 45 min in the reaction buffer (50 mM Tris-HCl, 150 mM NaCl, 20 mM $MgCl_2$). The concentrations used were 1 μM for each Twin-Strep-tag-fused bait peptide (1–3), 1 μM for GFP-MATH, 10 μM for SA-Pup^E, 10 mM for PafA12KR and 5 mM for ATP. Reactions were terminated by adding SDS loading buffer and boiling at 95°C for 10 min. The supernatant was then collected by centrifugation for WB detection.

For the conventional APPLE assay to identify PPIs, 3mg of cell lysate transfected with plasmid expressing the Twin-Strep-tag-fused bait protein was incubated in reaction buffer at 37°C for 45 min. The final concentrations of reagents were 0.25 μM SA-Pup^E, 0.25 μM PafA12KR and 5 mM ATP, with 0.5 mM PMSF added to prevent protein degradation. For mass spectrometry, reactions were stopped by adding 8M urea. The cell lysate was incubated with continuous rotation at room temperature until complete urea dissolution. 100 μL biotin agarose (Sigma-Aldrich) was added to the supernatant and incubated at 4°C overnight.

For APPLE assay on the cell surface, the culture medium was first removed from the dish, and cells were washed twice with pre-warmed PBS (37°C). A reaction mixture containing 0.2 μM GLP-1-Twin-Strep-tag, 0.2 μM SA-Pup^E and reaction buffer supplemented with 0.5mM PMSF was prepared in a 15 mL conical tube and incubated at room temperature for 30 min. This mixture was then added to cells in dish and incubated at 4°C for 1–2 h. Finally, 0.2 μM PafA and 5 mM ATP were added to initiate the enzymatic reaction, which proceeded at 37°C for 40 min. All steps were performed under sterile conditions with PMSF added immediately before use to maintain protease inhibition activity. After reaction completion, cells were lysed using RIPA buffer. After complete cell lysis, 8 M urea was added to the lysate to a final concentration of 8 M. The protein interaction complexes were then isolated using the same affinity capture procedure described above.

Immunoblotting

Following electrophoretic separation on 7.5–12.5% gradient SDS-polyacrylamide gels, proteins were transferred to methanol-activated PVDF membranes (Merck Millipore) using a wet transfer system (Mini Trans-Blot, Bio-Rad). The transfer buffer (25 mM Tris-HCl, 190 mM glycine, 20% methanol) was maintained at room temperature during 1.5 h transfer at 160 mA constant current. Membranes were then briefly rinsed with TBST and blocked for 2 h at room temperature with 5% non-fat dry milk (Sangon Biotech) in TBST. All primary and secondary antibodies (detailed in key resources table) were diluted in TBST and applied with three TBST washes between incubations. The membrane was scanned on an imager system (Typhoon).

Immunofluorescence

After a 48 h post-transfection, cells were washed twice with pre-warmed PBS and fixed with 4% paraformaldehyde (PFA) for 15–20 min at room temperature (protected from light). After three PBS washes, cells were permeabilized with 0.2% Triton X-100 in PBS for 10 min at room temperature and washed again three times with PBS. Non-specific binding was blocked overnight with 5% BSA in PBS at 4°C. Cells were then sequentially incubated with primary antibodies and corresponding fluorescent secondary antibodies, with thorough PBS washes between each step. Nuclei were counterstained with DAPI-containing anti-fade mounting medium, and coverslips were sealed with nail polish. Fluorescence images were acquired using a confocal microscope.

CellPress
OPEN ACCESS

Affinity purification for protein-protein interactions

30 μL STarm Streptactin Beads 4FF (Smart-Lifesciences Biotechnology, SA053005) were added to 3 mg cell lysate transfected with plasmid expressing the Twin-Strep-tag-fused bait protein in binding buffer (50 mM Tris-HCl, 150 mM NaCl, 20 mM $MgCl_2$, pH 7.4) and incubated overnight at 4°C with rotation. After incubation, beads were washed twice with PBS to remove nonspecifically bound proteins. The washed beads were then directly processed for mass spectrometry.

Poly (I:C) treatment

For Poly I:C transfection, Lipofectamine 3000 Transfection Reagent (Thermo Fisher Scientific, L3000001) was employed according to the manufacturer's protocol with modifications. Briefly, for each 10 cm culture dish, 24 μg of Poly I:C (Medlife, PC11566) was diluted in 500 μL of serum- and antibiotic-free DMEM medium and mixed gently by pipetting, followed by addition of 36 μL P3000 Reagent. In parallel, 48 μL of Lipofectamine 3000 reagent was diluted in another 500 μL of serum-free DMEM. The two solutions were combined within 5 min, mixed thoroughly, and incubated at room temperature for 15 min to allow complex formation. The resulting transfection complexes were then added dropwise to 293T cells that had reached approximately 85% confluency.

Coimmunoprecipitation

Protein-protein interactions were investigated through co-immunoprecipitation assays using HEK293 cells transiently expressing ORF9B-Twin-Strep-tag and HA-tagged candidate interactors. Following cell lysis, cleared supernatants containing 2 mg total protein were subjected to affinity purification using STarm Streptactin Beads 4FF (Smart-Lifesciences Biotechnology, SA053005) overnight at 4°C. After two washes with PBS, bound proteins were eluted by heat denaturation in SDS loading buffer (95°C, 10 min) and analyzed by immunoblotting with anti-strep-tag and anti-HA antibodies.

Mass spectrometry

Biotin-agarose enriched proteins were subjected to reduction with 10 mM DTT (37°C, 1 h) followed by alkylation using 25 mM iodoacetamide (dark incubation, 20 min). On-bead tryptic digestion was then performed at 37°C overnight using a 1:30 (w/w) enzyme-to-protein ratio. After digestion, the beads were washed twice with 200 μL of 50 mM ammonium bicarbonate (NH_4HCO_3). The combined supernatants containing tryptic peptides were desalted using Empore StageTips6094 SDB-RPS (MGAM Institute, YW-SDB-RPS-96) following the manufacturer's protocol.

The peptides were re-dissolved in solvent A (A: 0.1% formic acid in water) and analyzed by Orbitrap Exploris 480 coupled to an EASY-nanoLC 1200 system (Thermo Fisher Scientific, MA, USA). 3 μL peptide sample was loaded onto a 25 cm analytical column (75 μm inner diameter, 1.9 μm resin (Dr Maisch)) and separated with 60 min-gradient starting at 4% buffer B (80% ACN with 0.1% FA) followed by a stepwise increase to 50% in 53 min 40 s, 95% in 40 s and stayed there for 5 min 40 s. The column flow rate was maintained at 300 nL/min with the column temperature of 40°C. The electrospray voltage was set to 2 kV. The mass spectrometer was run under data independent acquisition (DIA) mode, and automatically switched between MS and MS/MS mode. The survey of full scan MS spectra (m/z:350–1200) was acquired in the Orbitrap with 120,000 resolutions. The Normalized automatic gain control (AGC) target of 300% and the maximum injection time of 50 ms. Then the precursor ions were selected into collision cell for fragmentation by higher-energy collision dissociation (HCD), the normalized collection energy was: 25%, 30%, 35%. The MS/MS resolution was set at 30,000, the Normalized automatic gain control (AGC) target of 200%, the maximum injection time of 50 ms.

Proteomics data analysis

Tandem mass spectra were processed by PEAKS Studio version 12 (Bioinformatics Solutions Inc., Waterloo, Canada). The databases were uniprot-homo_sapiens (version 2024, 20608 entries) and uniport-Rattus_norvegicus(version 2024, 22897 entries) which download from uniprot. Trypsin and Lys C was set as the digestion enzyme and semi-Specific was specified as the digest type. PEAKS DB were searched with a fragment ion mass tolerance of 0.02 Da and a parent ion tolerance of 10 ppm. The max missed cleavages was 2. The proteins with 1%FDR and containing at least 1 unique peptide were filtered.

In our experiments, the contaminant standards were proteins that appeared in >50%–70% of AP-MS experiments in the CRAPome database (version 2.0). Putative ORF9B interactors were clustered based on their kinetic profiles using Mfuzz time-course analysis. Protein interaction data were integrated from two public databases: BioGRID (version 4.4.244) for curated interactions and STRING (version 12.0) with a medium-confidence threshold (0.4). These datasets were used to generate a comprehensive cell membrane interactome map through Cytoscape platform (version 3.10.1).

Generation of endogenous tagging of PIN1 knock-in cell line

Addition of PIN1 gene endogenously labeled tag sequence within the genome of HEK293T cells was performed using CRISPR/Cas9 gene editing. Guide RNAs(gRNAs) were designed and cloned into a Cas9-mCherry expression vector to target the insertion site. For the donor construct, 800 base pair homology arms that span left and right of the start codon of PIN1 tagging were generated by Gibson assembly. HEK293T cells were plated in 6-well plates to reach 70% confluence on the next day for transfection. The gRNA plasmid (1.5μg) and donor construct (1.5μg) were introduced into HEK293T cells per well. After 48h of transfection, mCherry positive cells were sorted into 96 well plates to generate single cell clones. To confirm HDR knock-in, monoclonal cell lines were

genotyped via the gDNA extraction and PCR amplification. For wild type cells, the band corresponds to a 1010bp product, whereas the insertion of Tag results in a 1142bp product.

Structure analysis

The protein structures were retrieved from the Protein DataBank (PDB; https://www.rcsb.org/). Structural examinations, including surface representation and electrostatic potential analysis, were executed using PyMOL Molecular Graphics System (Version 4.6.0, Schrödinger LLC) with default rendering settings.

QUANTIFICATION AND STATISTICAL ANALYSIS

All statistical analyses were performed using GraphPad Prism (version 10.2.0). The number of replicates (n) of each experiment can be found in the respective figure caption, and n represents the number of independent shake flask cultures. Quantitative data were processed using ImageJ and MaxQuant, with normalization to appropriate controls. Multiple testing corrections (Benjamini-Hochberg FDR) were applied where appropriate, with significance thresholds set at $p < 0.05$. Significant m/z features were defined with a 1.5-fold cut-off and a p-value < 0.05 for a two-sample t-test.

Cell Reports Methods, Volume *5*

Supplemental information

Sensitive and specific affinity purification-mass spectrometry assisted by PafA-mediated proximity labeling

Shihan Luo, Lijuan Xie, Lin Yang, Zheyao Hu, Lei Wang, Yueqin Wang, Qingqing Li, Shujuan Guo, Shengce Tao, and Hewei Jiang

Figure S1

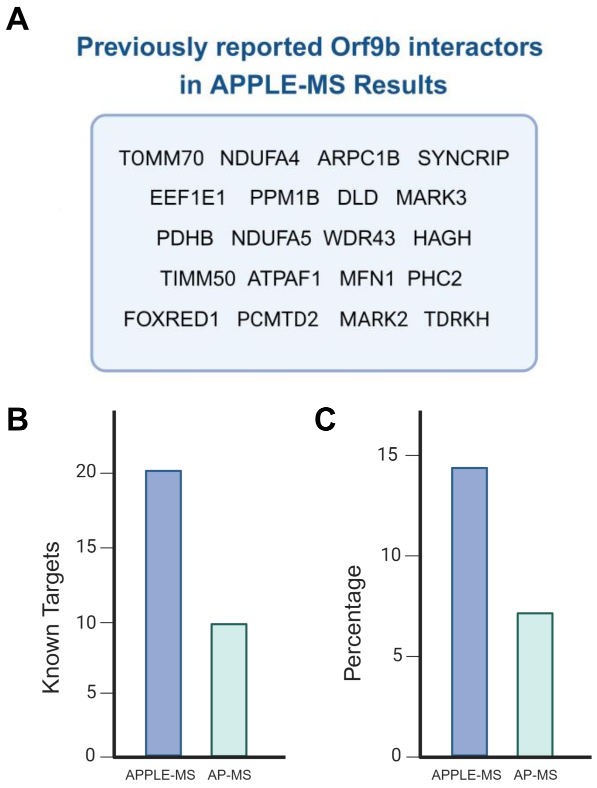

Figure S1 Comparative analysis of ORF9B interactome detection by APPLE-MS versus AP-MS, related to Figure 2.

(A) Reported ORF9B interactors from BioGRID (v4.4.244) detected by APPLE-MS

(B) Quantitative assessment of reported ORF9B interactors. Bar plot illustrates the absolute number of benchmark ORF9B interactors identified by APPLE-MS (blue) versus conventional AP-MS (green).

(C) Detection efficiency for reported ORF9B interactors. Bar plot shows the percentage of known interactors among total identified putative binding partners for each method.

Figure S2

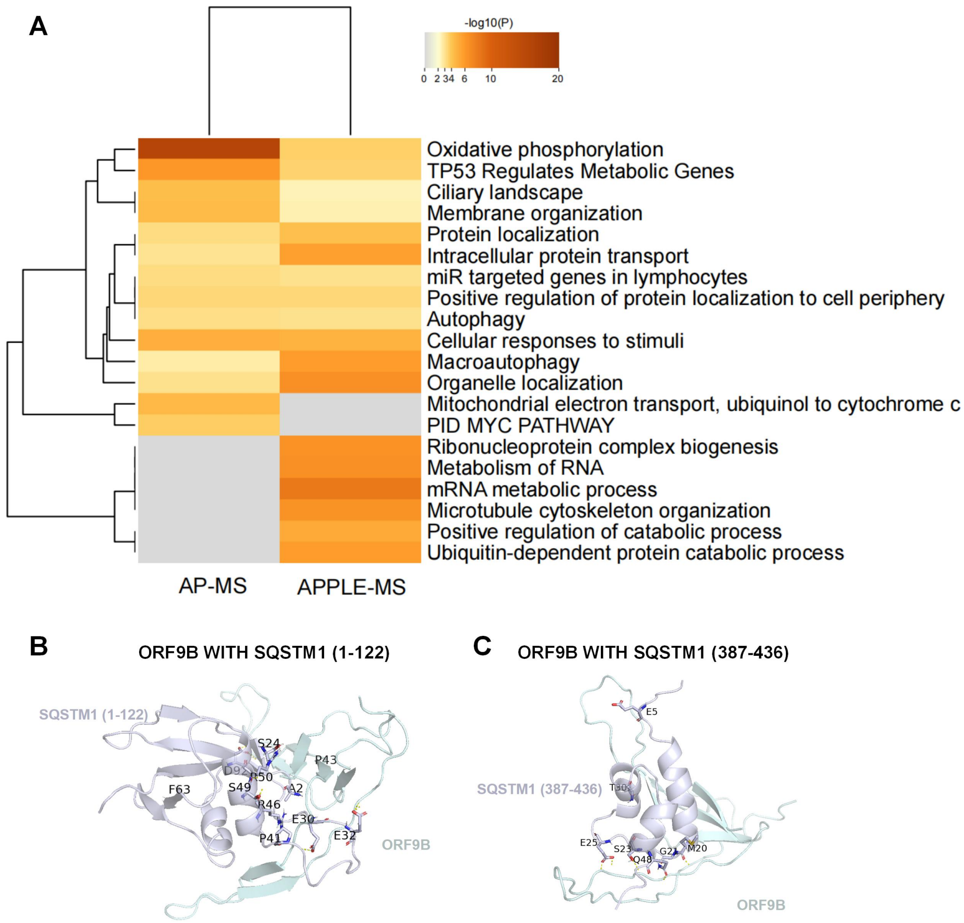

Figure S2 Comparative interactome analysis and structural modeling of ORF9B~ SQSTM1 complexes, related to Figure 3.

(A) GO enrichment comparison of ORF9B interactors between AP-MS (left) and APPLE-MS (right). Color intensity indicates -log10(p value).

(B) and (C) Predicted interaction interfaces between ORF9B and SQSTM1. Molecular dynamics simulations of ORF9B (cyan) binding to distinct SQSTM1 domains (purple).

Figure S3

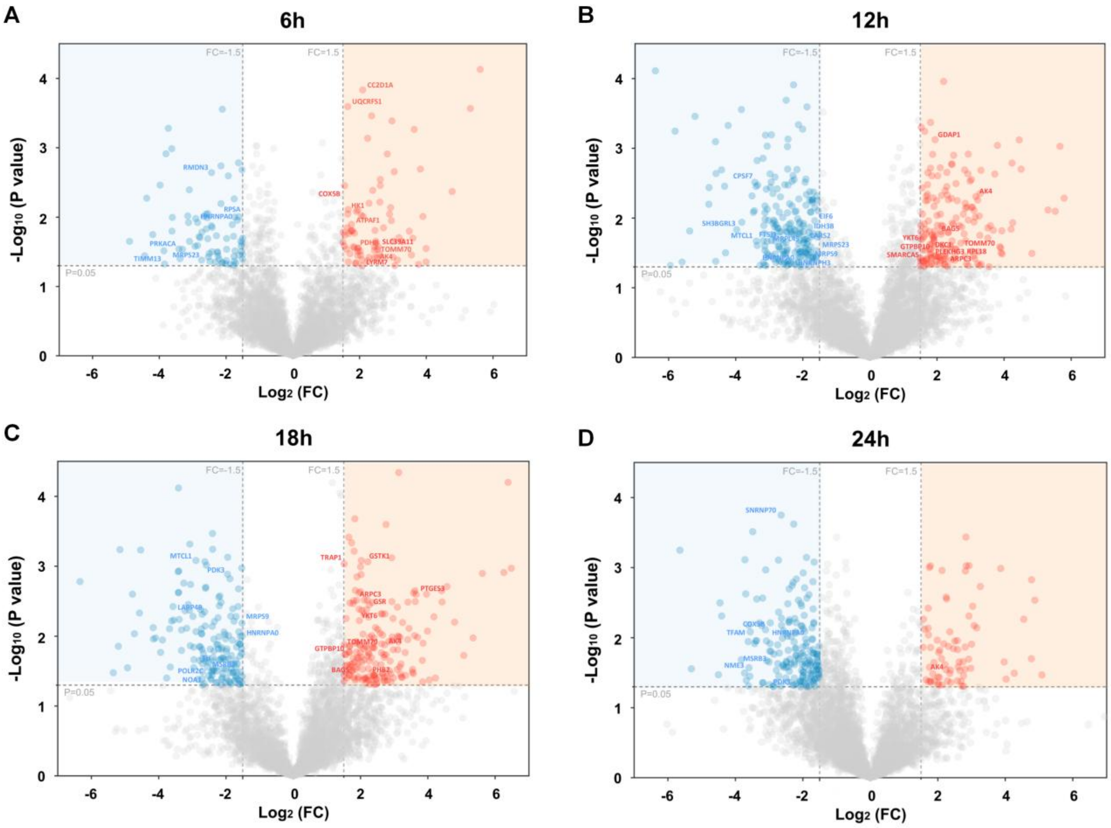

Figure S3 Temporal Profiling of the ORF9B Interactome Following Poly(I:C) Transfection, related to Figure 4.

(A-D) Time-resolved analysis of ORF9B-protein interactions after poly(I:C) transfection. Volcano plots display significantly enriched interactors ($p < 0.05$, fold-change > 2) at each time point (6, 12, 24, and 48 h; n = 3 biological replicates). Literature-curated ORF9B interactors from BioGRID (v4.4.244) are highlighted in red. Dashed lines indicate statistical thresholds (horizontal: $p = 0.05$; vertical: 1.5-fold change).

Figure S4

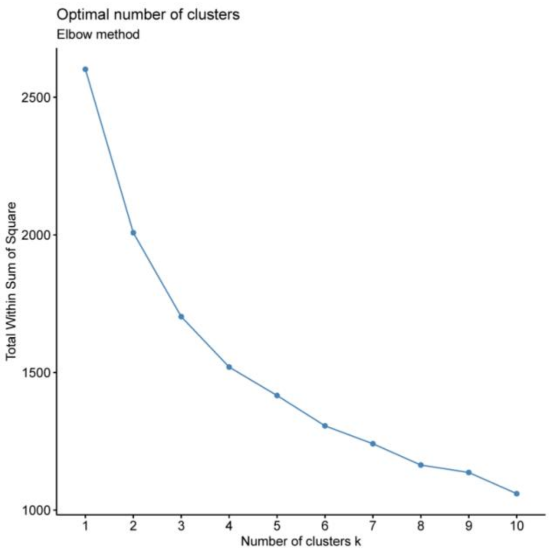

Figure S4 Determination of Optimal Cluster Number in Mfuzz Analysis, related to Figure 4.

Line plot of SSR values versus cluster number (k = 1–10) computed from Mfuzz soft clustering of ORF9B interactome dynamics (0–24 h poly(I:C)). The elbow point at k = 4 was selected as the optimal cluster number, balancing model fit and biological interpretability.

Figure S5

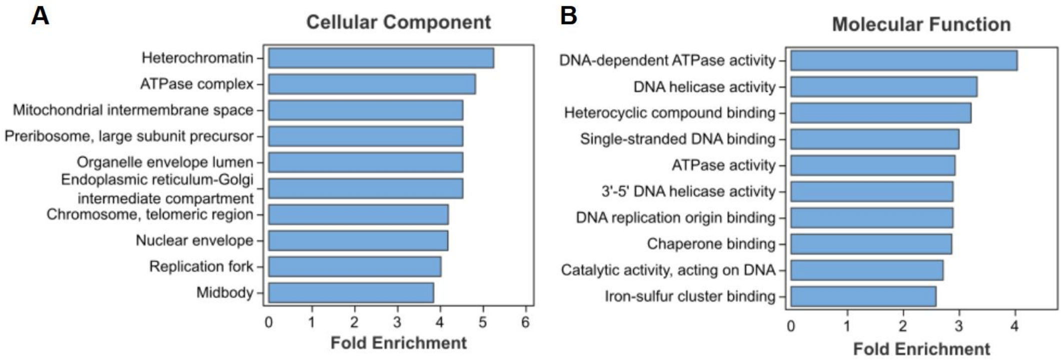

Figure S5 Cellular component and molecular function of PIN1 putative interactors, related to Figure 5.

(A) Subcellular localization of ORF9B interactome. Bar plot shows significantly enriched cellular components (FDR<0.05, hypergeometric test) for ORF9B-binding partners.

(B) Molecular functions enriched in ORF9B interactors. Bar plot illustrates significantly enriched molecular functions (FDR<0.05, hypergeometric test) among ORF9B-associated proteins identified by APPLE-MS.

In (A) and (B), the length of each bar corresponds to the $-\log_{10}$(FDR) value, representing the statistical significance of enrichment.
